# Sex-stratified genome-wide association study of multisite chronic pain in UK Biobank

**DOI:** 10.1371/journal.pgen.1009428

**Published:** 2021-04-08

**Authors:** Keira J. A. Johnston, Joey Ward, Pradipta R. Ray, Mark J. Adams, Andrew M. McIntosh, Blair H. Smith, Rona J. Strawbridge, Theodore J. Price, Daniel J. Smith, Barbara I. Nicholl, Mark E. S. Bailey

**Affiliations:** 1 Institute of Health and Wellbeing, University of Glasgow, Glasgow, Scotland, United Kingdom; 2 Division of Psychiatry, University of Edinburgh, Edinburgh, Scotland, United Kingdom; 3 School of Life Sciences, College of Medical, Veterinary & Life Sciences, University of Glasgow, Glasgow, Scotland, United Kingdom; 4 School of Behavioral and Brain Sciences, The University of Texas at Dallas, Richardson, Texas, United States of America; 5 Division of Population Health Sciences, University of Dundee, Ninewells Hospital and Medical School, Dundee, Scotland, United Kingdom; 6 Department of Medicine Solna, Karolinska Institute, Stockholm, Sweden; Emory University, UNITED STATES

## Abstract

Chronic pain is highly prevalent worldwide and imparts a significant socioeconomic and public health burden. Factors influencing susceptibility to, and mechanisms of, chronic pain development, are not fully understood, but sex is thought to play a significant role, and chronic pain is more prevalent in women than in men. To investigate sex differences in chronic pain, we carried out a sex-stratified genome-wide association study of Multisite Chronic Pain (MCP), a derived chronic pain phenotype, in UK Biobank on 178,556 men and 209,093 women, as well as investigating sex-specific genetic correlations with a range of psychiatric, autoimmune and anthropometric phenotypes and the relationship between sex-specific polygenic risk scores for MCP and chronic widespread pain. We also assessed whether MCP-associated genes showed expression pattern enrichment across tissues. A total of 123 SNPs at five independent loci were significantly associated with MCP in men. In women, a total of 286 genome-wide significant SNPs at ten independent loci were discovered. Meta-analysis of sex-stratified GWAS outputs revealed a further 87 independent associated SNPs. Gene-level analyses revealed sex-specific MCP associations, with 31 genes significantly associated in females, 37 genes associated in males, and a single gene, *DCC*, associated in both sexes. We found evidence for sex-specific pleiotropy and risk for MCP was found to be associated with chronic widespread pain in a sex-differential manner. Male and female MCP were highly genetically correlated, but at an r_g_ of significantly less than 1 (0.92). All 37 male MCP-associated genes and all but one of 31 female MCP-associated genes were found to be expressed in the dorsal root ganglion, and there was a degree of enrichment for expression in sex-specific tissues. Overall, the findings indicate that sex differences in chronic pain exist at the SNP, gene and transcript abundance level, and highlight possible sex-specific pleiotropy for MCP. Results support the proposition of a strong central nervous-system component to chronic pain in both sexes, additionally highlighting a potential role for the DRG and nociception.

## Author summary

Chronic pain is a highly prevalent and debilitating condition, which is more common in women than in men. Sex differences in this condition may be a result of several factors, including differences between the sexes in genetic variation related to chronic pain and gene expression differences related to sex. To explore sex differences in chronic pain from a genetic perspective, we looked for genetic variants associated with chronic pain in men and women separately in a large general-population cohort, and compared the variants we identified between the sexes. We assessed the degree of overlap between genetic variants associated with chronic pain in each sex and those associated with a wide range of other traits, including major depression, body-mass index and suicidality. We also investigated gene expression patterns across a range of tissues for genes associated with chronic pain in each sex, in particular examining expression in neural and non-neural human and mouse tissues and assessing the degree of Dorsal Root Ganglion (DRG) enrichment, an important peripheral nervous system component involved in chronic pain. This work contributes to understanding of chronic pain as a trait and of sex differences in chronic pain at the levels of genetics and gene expression.

## Introduction

Chronic pain is widely defined as pain persisting beyond 3 months [1,2], and can be a primary disorder [3] or secondarily associated with injury, surgery or a range of medical conditions. Chronic pain is highly prevalent worldwide [4–9] and imparts a significant socioeconomic and public health burden [10]. Factors influencing susceptibility to chronic pain, and the mechanisms underlying its development and maintenance, are not fully understood.

Several aspects of chronic pain including Chronic Pain Grade [11], severe chronic pain and low back pain have been studied from a genetic perspective and found to be complex traits. Heritability estimates vary from ~30–46% in twin, pedigree and factor analysis studies [12–15], while single nucleotide polymorphism (SNP) heritability has been estimated from genome-wide association studies (GWAS) to be ~7–10% [16,17].

It is increasingly recognised that sex differences in many complex human traits are biologically important, with genetic architecture for many traits being to some extent sex-specific [18], and a ‘sex-aware’ approach to genetic analysis has been widely advocated [19]. Sex as a biological variable has wide-ranging effects on the functioning of the genome and on resultant phenotypes. These effects can be mediated via sex-differential gene expression [20,21], sex differences in methylation [22–26] and expression quantitative trait locus (eQTL) effects [27,28], or differing levels and actions of hormones [29,30]. Sex can also influence traits through environmental factors strongly correlated with sex [23,24] and sex-specific pleiotropy [25,31]. Chronic pain exhibits sex-related prevalence differences, and is more common in women than in men [32–34]. There are also potential sex differences in the impact of pain on functioning in daily life, and in the success of specific coping strategies [35]. In addition to differences in prevalence between the sexes, sex differences in underlying pain mechanisms and their modulation by immune cells have been recently reported [36–38], and immune responses in general can differ by sex [39].

Multisite Chronic Pain (MCP) is a derived chronic pain phenotype, defined as the sum of the number of sites of chronic pain on the body, here expressed on a scale from 0–7 [17]. We have previously shown in UK Biobank [17] that genetic predisposition to MCP (as captured by a polygenic risk score; PRS) was associated with Chronic Widespread Pain (CWP), a separate but related chronic pain phenotype, in women but not men [17]. Additional unpublished findings using the Generation Scotland study [40] demonstrated that the PRS-MCP was associated with both chronic pain grade and an MCP-like phenotype in both men and women, but that the magnitude of effect was roughly twice as great in women as it was in men. There was also a significant PRS-by-sex interaction. These findings suggest that sets of variants contributing to chronic pain in males and females may act differently, or have different genetic effect sizes, in the two sexes. Here we report on an exploration of this preliminary evidence for the existence of sex-specific loci associated with MCP using a sex-stratified GWAS analysis approach in UK Biobank, and identify several sex-specific MCP loci. A meta-analysis of the female- and male-specific GWASs also revealed novel MCP loci not identified in the original MCP GWAS. We have also investigated possible functional effects associated with sex-specific MCP-associated genes as revealed by gene expression data in multiple relevant tissues, including dorsal root ganglion (DRG) samples, in both human and mouse.

## Results

### GWAS of MCP in males and females separately

**Fig 1:**
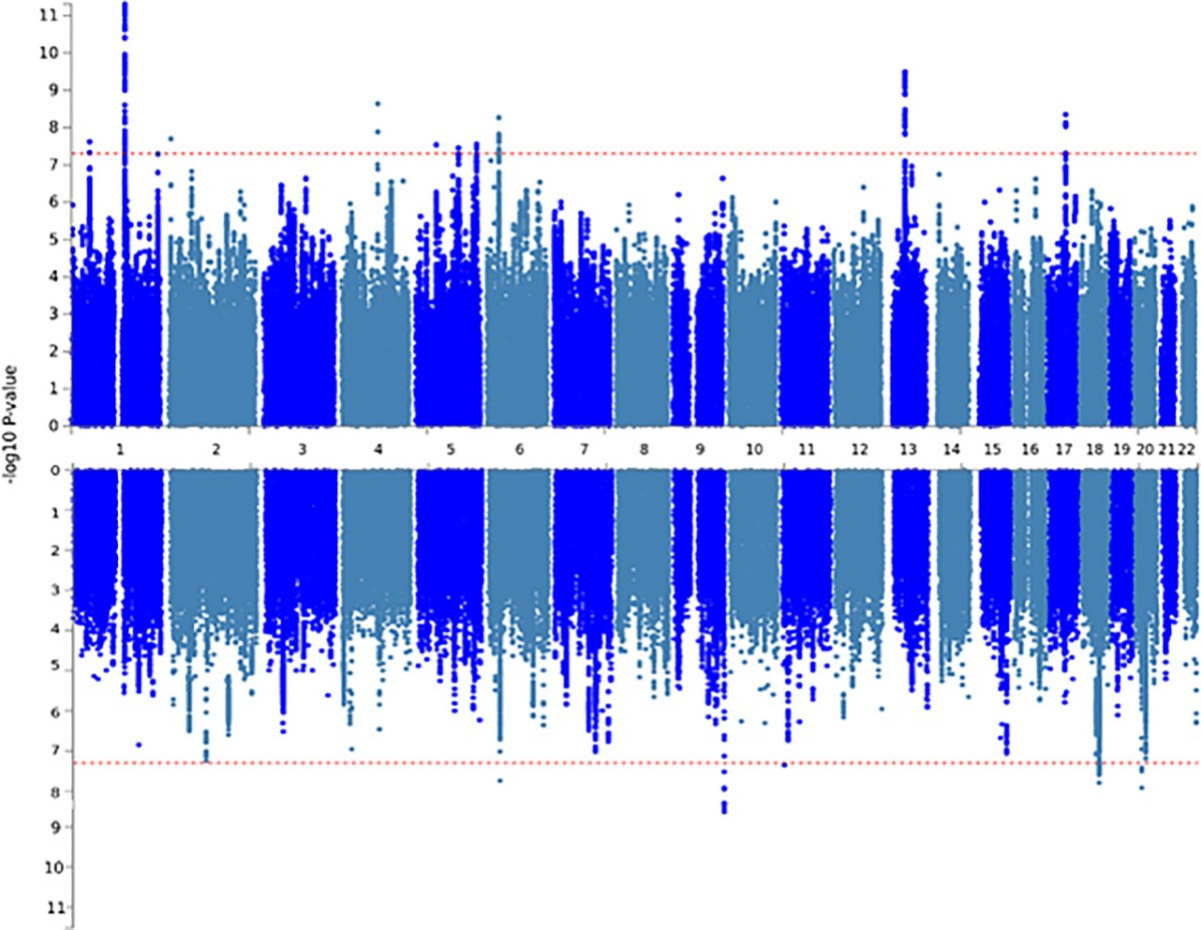
Manhattan plots for the stratified GWAS analyses. Upper panel: female, lower panel: male. Red dotted line indicates genome-wide significant p-value threshold.

To detect sex-differential genetic influences on multisite chronic pain, GWASs were run separately for males and females in UK Biobank. In men, a total of 123 SNPs at five independent loci were associated with MCP at a genome-wide significance threshold of p < 5 x 10^−8^ (Table 1, Fig 1). In women, a total of 286 genome-wide significant SNPs at ten independent loci were discovered (Table 1, Fig 1). All 15 of these loci were differentially associated with sex—none of the genome-wide significant SNPs at these loci had p < 5 x 10^−8^ in the GWAS conducted in the opposite sex. However, a total of 257 SNPs were found to have suggestive levels of association with MCP (p < 5 x 10^−5^) in both men and women. Two SNPs had suggestive evidence in men and were genome-wide significant in women, and eight SNPs had suggestive evidence in women and were genome-wide significant in men. In addition, the genome-wide significant loci on chromosome 6 in each sex were separated by less than 1 Mbp and may potentially exert their influence via differential effects on the same gene.

**Table 1:** Genome-wide significant independent lead SNPs associated with MCP (p < 5 x 10^−8^) in male and female sex-stratified GWAS. CHR, chromosome; BP, chromosome coordinate; A1/A2, alleles 1 and 2, where A1 is the effect allele; BETA/SE, coefficient and its standard error for the effect allele; Gene = ANNOVAR annotation gene symbol for variant (N/A, no gene annotation in ANNOVAR).

SNP rsID	CHR	BP	A1	A2	BETA	SE	P	Sex	Gene
rs35072907	1	51189556	G	C	0.020	0.004	2.40E-08	Female	FAF1
rs59898460	1	150493004	T	C	0.025	0.004	4.90E-12	Female	LINC00568; RP11-54A4.2
rs147903676	2	5835352	C	CT	-0.031	0.006	2.00E-08	Female	SOX11
rs13135092	4	103198082	A	G	-0.038	0.006	2.30E-09	Female	SLC39A8
rs3080367	5	57576558	TACAC	T	0.024	0.004	2.90E-08	Female	PGAM1P1; PLK2
rs62381120	5	120176330	T	C	-0.021	0.004	3.50E-08	Female	CTD-2334D19.1; AC008565.1
rs74274428	5	170842428	CA	C	0.020	0.004	2.80E-08	Female	NPM1; FGF18
rs151060048	6	34633069	CA	C	-0.035	0.006	5.40E-09	Female	C6orf106
rs34003284	13	53902876	C	A	-0.024	0.004	3.20E-10	Female	RN7SL618P; AL450423.1
rs11079993	17	50301552	G	T	-0.021	0.004	4.50E-09	Female	CA10; snoZ178
rs10660361	6	33741371	C	CG	0.020	0.004	1.80E-08	Male	LEMD2
9:140251458_G_A	9	140251458	G	A	-0.030	0.005	3.00E-09	Male	EXD3
rs16909443	11	6192462	T	C	-0.040	0.007	4.40E-08	Male	RP11-290F24.3
18:50442591_TTTC_T	18	50442591	TTTC	T	-0.020	0.004	1.60E-08	Male	N/A
20:19709268_AAAAT_A	20	19709268	AAAAT	A	0.030	0.005	1.20E-08	Male	SLC24A3; AL121761.1

LDSR analysis demonstrated that inflation of test statistics in each GWAS was due to polygenicity (Table 2; LDSR intercept). SNP heritability was moderate, estimated as 0.125 and 0.106 in females and males, respectively (Table 2). The genetic correlation between male and female MCP was high (r_g_ = 0.92, SE 0.03; p = 3.32 x 10^−213^), but significantly less than 100% (based on confidence intervals calculated as +/- 2 x SE).

**Table 2:** Trait genetic attributes from the male and female MCP GWASs. SNP-heritability, BOLT-LMM pseudo-heritability estimate; λ_GC1000_, λ_GC_ value adjusted for sample size; LDSR_intercept (SE), LD-score regression intercept value and its standard error.

Attribute	female	male
SNP-heritability	0.125	0.106
λ_GC1000_	1.002	1.001
LDSR_intercept (SE)	1.03 (0.006)	1.025 (0.005)

### Meta-Analysis of Sex-Stratified MCP GWAS Outputs

87 independent SNPs were found to be associated with MCP at genome-wide significance in total, 11 of which were novel (not found in the unstratified or in each sex-stratified GWAS analysis). Each of the 87 independent significant SNPs showed consistent direction of effect between males and females (Table 3), but seven showed significant heterogeneity in effect size (I^2^ p < 0.05). In total, 49 lead SNPs across 46 genomic risk loci were found to be associated with MCP in meta-analysis of sex-stratified outputs.

**Table 3:** Independent, genome-wide significant (GWS) SNPs from meta-analysis of sex-stratified MCP GWASs. Genomic Locus = genomic risk locus context of SNP with multiple independent GWS SNPs present at some of the 46 loci, rsID = SNP rsID identifier, Position = genomic position (chromosome: base-pair start position), Meta p = p value for association from the GWAS meta-analysis, Direction = direction of effect in female MCP and male MCP GWASs, respectively (+ if association beta value for effect allele > 0,—if < 0), I^2^ = effect size heterogeneity estimate, I^2^ p = p value for heterogeneity estimate, Symbol = ANNOVAR gene annotation for variant (N/A, no gene annotation in ANNOVAR). Significant (unadjusted) heterogeneity I^2^ p values (I^2^ p < 0.05) are marked with *.

rsID	Genomic Locus	Position	Meta p	Direction	I^2^	I^2^ p	Symbol
rs909001	1	1:32196647	2.80E-08	--	7.6	0.298	BAI2
1:51042504_CT_C	2	1:51042504	2.35E-08	--	59.1	0.118	FAF1
rs197441	3	1:112283655	4.33E-10	--	0	0.644	FAM212B-AS1
rs12033257	3	1:112318484	2.79E-08	++	0	0.824	KCND3
rs509345	4	1:150276022	1.42E-10	--	71.3	0.062	MRPS21
rs367563576	4	1:150495378	2.24E-11	++	89.6	0.002*	LINC00568; RP11-54A4.2
rs9700909	5	1:243255124	4.07E-09	--	30	0.232	RP11-261C10.3
1:243461350_CT_C	5	1:243461350	9.48E-09	--	53	0.145	SDCCAG8
rs6721975	6	2:5832667	2.86E-08	--	67	0.082	SOX11
rs4852567	7	2:80703379	3.51E-08	++	25	0.248	CTNNA2
rs5832889	8	2:100503396	2.06E-08	++	74.9	0.046*	AFF3
rs112908707	9	3:49865628	4.35E-10	--	0	0.866	TRAIP
rs62260755	9	3:49898318	5.36E-09	--	0	0.619	CAMKV
3:50098024_CAA_C	9	3:50098024	4.79E-09	++	0	0.75	RBM6
rs13067082	9	3:50221715	3.66E-08	++	0	0.534	SEMA3F
rs144433312	10	3:84591507	1.07E-08	++	26.7	0.243	AC107025.1; LINC00971
rs62263345	11	3:107252190	5.01E-10	--	0	0.848	BBX
rs28750366	12	3:136361055	3.55E-08	--	0	0.498	STAG1
rs56203712	13	4:25342606	1.37E-11	++	0	0.783	ZCCHC4
rs201081507	14	4:102681041	8.93E-09	--	51.9	0.149	BANK1
rs13109404	14	4:102896591	1.17E-09	--	0	0.768	BANK1
rs13135092	14	4:103198082	2.61E-14	--	3.4	0.309	SLC39A8
rs6869446	15	5:65570607	3.23E-08	--	3.4	0.309	snoU13; RP11-305P14.1
rs10076888	16	5:103786487	1.06E-08	++	0	0.872	RP11-6N13.1
rs147831713	16	5:103787168	9.96E-09	--	0	0.66	RP11-6N13.1
rs325485	16	5:103995368	3.15E-08	++	0	0.576	RP11-6N13.1
rs1976423	16	5:104042643	1.48E-08	--	58.3	0.121	RP11-6N13.1
rs137863733	17	5:160890323	8.13E-10	++	23.3	0.254	GABRB2
rs6915136	18	6:33651322	1.09E-08	--	37.2	0.207	ITPR3
rs482786	18	6:33707599	1.51E-09	--	29.8	0.233	IP6K3
6:33709752_CA_C	18	6:33709752	1.08E-08	++	0	0.5	IP6K3
rs28651968	18	6:33717424	2.16E-08	++	0	0.945	IP6K3; LEMD2
rs17529077	18	6:33793332	1.27E-09	++	0	0.997	MLN; LINC01016
rs17600945	18	6:33802263	1.21E-09	++	0	0.365	MLN; LINC01016
rs6907508	19	6:34592090	2.81E-08	--	50.3	0.156	C6orf106
rs151060048	19	6:34633069	6.92E-09	--	84.4	0.011*	C6orf106
rs142415291	19	6:34755312	9.43E-09	--	81.6	0.020*	SNRPC; UHRF1BP1
rs6926377	20	6:145105354	7.17E-09	--	49.6	0.159	UTRN
rs148148187	21	7:3602520	1.29E-08	++	0	0.839	SDK1
rs7798894	22	7:21552995	3.44E-08	++	17	0.273	SP4
rs6966540	23	7:95727967	1.09E-08	--	0	0.67	DYNC1I1
rs10156143	23	7:95844896	6.43E-09	--	0	0.349	SLC25A13
rs1450833	24	7:113865735	1.16E-08	--	0	0.715	FOXP2
7:113945981_CCACTTATAAATACTGTCCCTTGGGCA_C	24	7:113945981	1.24E-08	++	0	0.657	FOXP2
rs1527146	24	7:113987281	4.56E-08	++	27.4	0.241	FOXP2
7:114058731_CA_C	24	7:114058731	5.08E-10	++	23.2	0.254	AC073626.2
rs55671932	25	7:150556803	4.07E-08	++	68.2	0.076	AOC1
rs6997840	26	8:141658361	2.97E-08	--	0	0.37	AGO2; PTK2
9:96168164_CT_C	27	9:96168164	1.83E-08	++	0	0.87	RNU6-829P; Y_RNA
rs7869969	27	9:96217447	9.87E-10	++	0	0.797	FAM120A
rs6478241	28	9:119252629	6.61E-09	++	5.6	0.303	ASTN2
9:140247497_A_C	29	9:140247497	5.65E-12	--	0	0.567	EXD3
9:140249861_A_C	29	9:140249861	6.70E-09	++	23.9	0.252	EXD3
9:140260266_T_G	29	9:140260266	3.38E-14	--	0	0.571	EXD3
rs2183271	30	10:21957229	4.47E-08	--	4.3	0.307	MLLT10
10:99784552_CCA_C	31	10:99784552	1.05E-08	--	40.4	0.195	CRTAC1
rs11599236	32	10:106454672	3.33E-08	++	0	0.351	SORCS3
rs17553733	33	11:16362089	1.70E-10	--	0	0.392	SOX6
rs2118362	33	11:16373083	1.32E-08	--	31.9	0.226	SOX6
rs55670730	34	11:43620008	4.18E-08	--	0	0.925	N/A
rs7303462	35	12:23974911	4.61E-08	--	32.5	0.224	SOX5
rs184483429	36	12:107620106	4.87E-08	++	0	0.58	RP11-797M17.1; SETP7
rs2759694	37	13:53695378	3.17E-08	++	76.8	0.038*	OLFM4; LINC01065
rs67128127	37	13:53889000	7.48E-09	++	3.4	0.309	RN7SL618P; AL450423.1
rs1443914	37	13:53917230	4.73E-10	++	76.7	0.038*	RN7SL618P; AL450423.1
rs7335163	37	13:53989975	7.23E-09	++	8.9	0.295	AL450423.1; LINC00558
rs17574479	37	13:54049489	2.97E-09	--	88.3	0.003*	AL450423.1; LINC00558
rs34521521	38	14:73832318	4.99E-08	--	0	0.512	NUMB
rs4886649	39	15:75328595	2.25E-08	--	0	0.892	PPCDC
15:75348905_CAACA_C	39	15:75348905	1.62E-08	--	0	0.924	PPCDC
rs2386584	40	15:91539572	6.68E-12	--	0	0.578	PRC1
rs285027	41	16:77100932	9.39E-09	--	0	0.748	CTD-2336H13.2; MON1B
rs11871043	42	17:43172849	1.49E-09	++	0	0.945	NMT1
rs967823	43	17:50317276	1.98E-11	--	58.8	0.119	snoZ178; RP11-429O1.1
rs35518690	44	18:42136963	2.15E-08	++	41.5	0.191	CTC-782O7.1; RP11-456K23.1
rs2043187	45	18:50394405	3.58E-10	--	0	0.668	DCC
rs72922230	45	18:50394407	4.12E-10	--	29.7	0.233	DCC
rs767443167	45	18:50622162	1.43E-08	--	0	0.469	DCC
rs8089828	45	18:50669725	9.63E-10	++	0	0.783	DCC
rs8099145	45	18:50743672	2.15E-12	--	0	0.871	DCC
rs12968428	45	18:50750225	1.77E-09	++	0	0.413	DCC
rs17410557	45	18:50776391	1.03E-11	--	0	0.561	DCC
rs773737322	45	18:50846440	9.82E-11	++	0	0.529	DCC
rs1367635	45	18:50861409	5.25E-09	--	0	0.659	DCC
rs766498304	45	18:50871256	7.87E-09	--	35.6	0.213	DCC
rs10164055	45	18:50919600	2.53E-08	--	0	0.659	DCC
rs16980973	46	20:19648493	1.51E-10	++	0	0.322	SLC24A3

### Genetic correlations between sex-stratified MCP and other disorders and traits

A range of complex trait phenotypes were selected for LDSR analysis with male and female MCP based on previous evidence for phenotypic correlation [17], with the addition of newly available trait data such as GWAS outputs on suicide and self-harm [41] and mood instability [42]. Suicidality and self-harm are important comorbidities of chronic pain, an issue compounded by the common co-occurrence of mental health traits, such as major depressive disorder (MDD), with chronic pain, and by the fact that use of certain medication in chronic pain contributes to increased risk for self-harm, suicidal ideation and suicide attempt [43–48].

**Fig 2:**
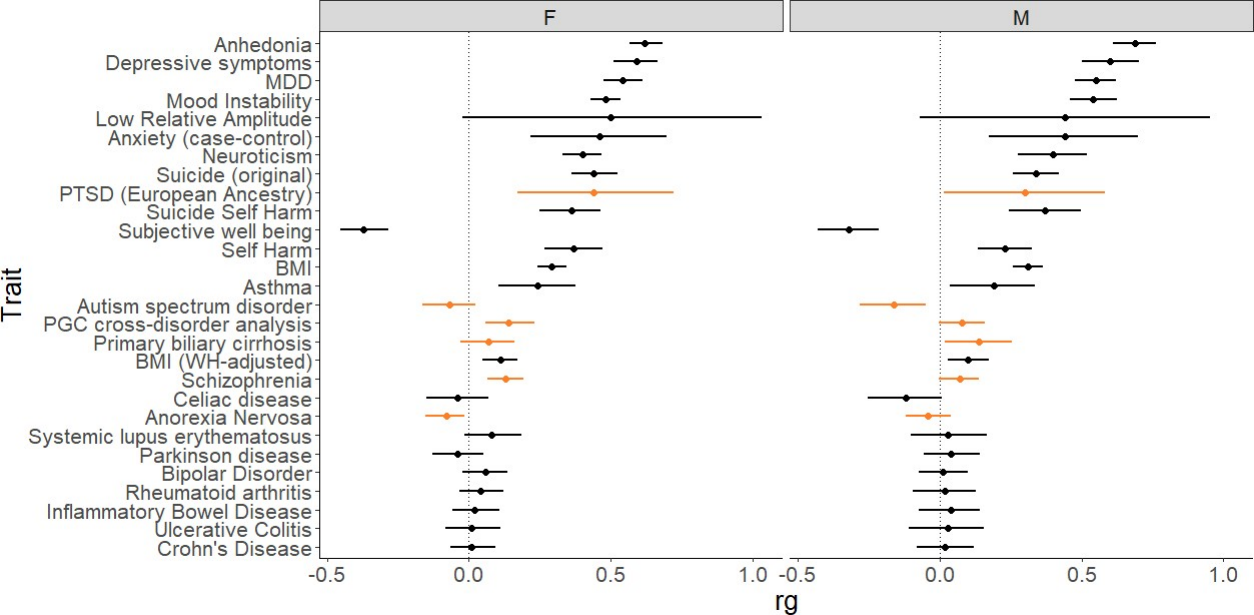
Genetic correlations (r_g_) between male (M) and female (F) MCP and multiple traits (r_g_ values with 95% CI error bars). Phenotypes differentially correlated with MCP between the sexes are plotted in orange.

MCP in both men and women was found to be significantly genetically correlated with a range of traits and disorders, including psychiatric and mood phenotypes such as anhedonia, mood instability, depressive symptoms, MDD, anxiety, suicidality and subjective wellbeing (Fig 2, S1 Table). In addition, as expected, genetic correlations between both male and female MCP and unstratified MCP from our previous analysis [17]were essentially perfect (r_g_ = 1.00, p < 1 x 10^−120^ for both sexes). PTSD, schizophrenia, autism spectrum disorder, anorexia nervosa, PGC cross-disorder phenotype and primary biliary cirrhosis were found to be significantly correlated with MCP in one sex and not the other. Several phenotypes were found not to be genetically correlated with MCP in either sex (p_fdr_ > 0.05), including inflammatory bowel diseases, Parkinson’s disease, bipolar disorder, rheumatoid arthritis, and low relative amplitude (a circadian rhythmicity-related phenotype).

### Gene-level analysis

**Fig 3:**
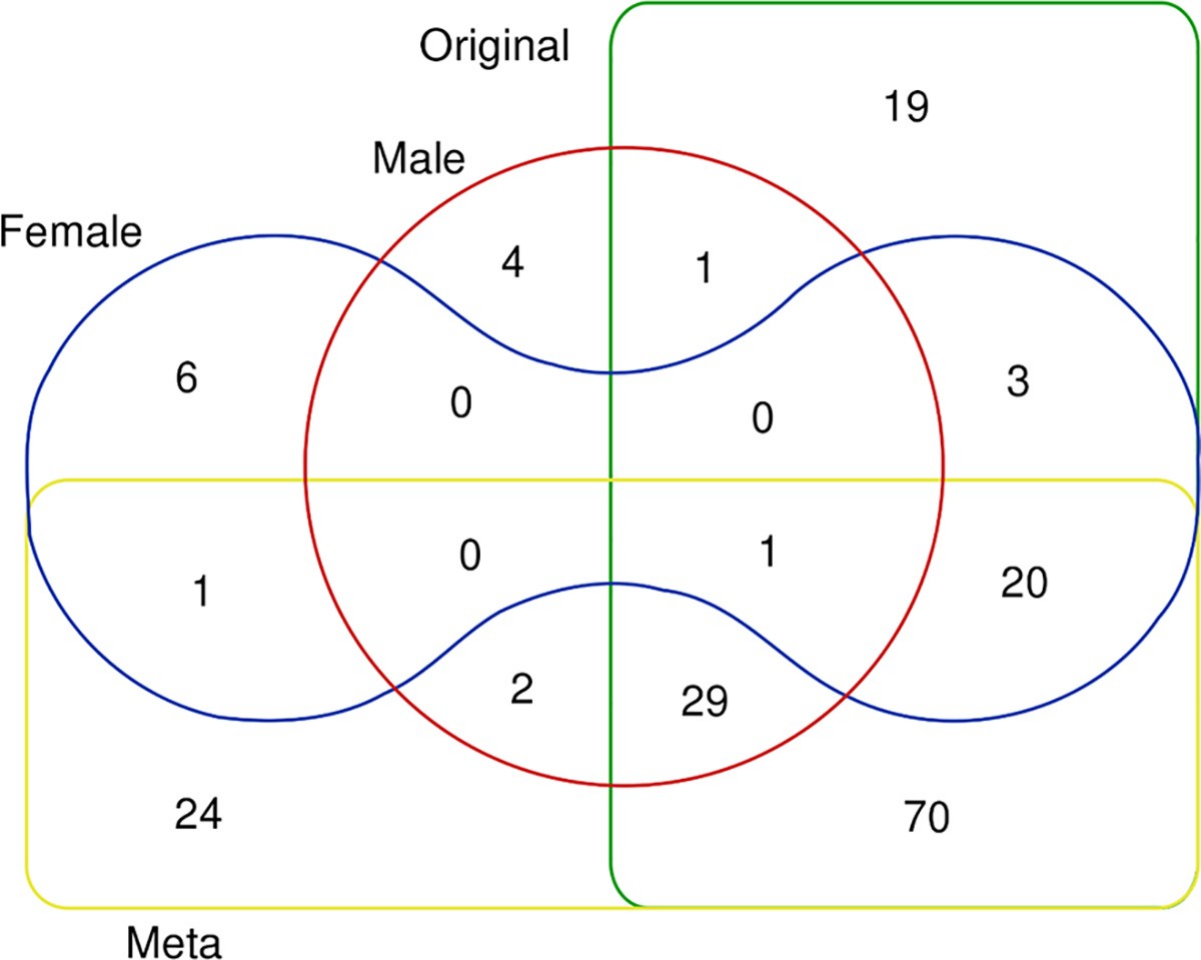
Venn diagram of number of genes found significantly associated with MCP in MAGMA gene-based test analyses. Sectors: Original = non-sex-stratified MCP GWAS, Meta = meta-analysed female and male GWAS output, Female = female MCP GWAS, Male = male MCP GWAS.

Genes enriched for variants associated with MCP were identified using a gene-level association analysis (gene-based test) [49] approach implemented by MAGMA as part of the FUMA suite that tests 19,012 separate genes. The results are summarised in Fig 3 and S2 Table. In females and males, 31 and 37 genes, respectively, were found to be significantly (Bonferroni-adjusted significance criterion: p < 2.63 x 10^−6^) associated with MCP. The only gene found to be significantly associated with both male and female MCP was *DCC*. 24 out of the 31 genes significantly associated with MCP in females in the sex-stratified analyses, and 31 out of the 37 genes significantly associated in males, were also significantly associated in our previous non-stratified analysis [17]. Six genes were significantly associated with MCP only in females (*NCAN*, *SPATS2L*, *TBC1D9*, *CAMK1D*, *SOX11*, *GON4L*), while 4 genes were associated only in males (*CENPW*, *MTCH2*, *NICN1*, *DNAJA4*). Twenty-four genes were identified as significantly associated with MCP only in the meta-analysis of the sex-stratified GWAS outputs (Fig 3 and S3 Table). Nineteen genes found in previous sex-combined GWAS analyses [17] were not found to be associated with MCP in either sex-stratified GWAS, or in GWAS meta-analysis (Fig 3 and S2 Table).

None of the six and four genes associated with MCP in females and males only respectively had a mouse orthologue listed in the Pain Genes Database [50], an interactive web browser listing mouse genes associated with pain-related phenotypes when knocked out. None of the six and four genes associated with MCP in females and males only respectively had a mouse ortholog listed in the Pain Genes Database [50], an interactive web browser of pain-related transgenic knockout studies. Of the twenty-four novel genes found to be associated with MCP in GWAS meta-analyses and associated downstream analyses, one had an ortholog listed in the Pain Genes Database (Gnaq)[51].

### Gene expression analysis

**Fig 4:**
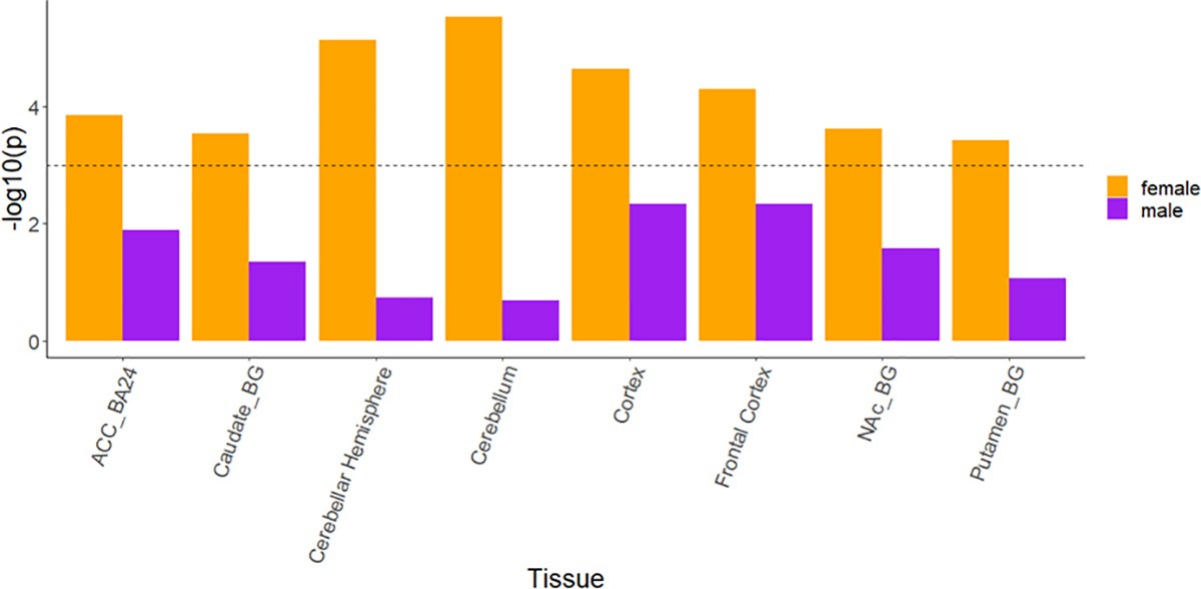
MCP-associated gene expression tissue enrichment analysis. Significant (-log_10_ p > 3) results in female analyses and their corresponding tissue results in male analyses are shown for brevity. No significant enrichment for expression in any tissue was found for MCP-associated genes in males. Dashed line = significance threshold. ACC_BA24 = anterior cingulate cortex BA24, Caudate_BG = caudate basal ganglia, NAc_BG = Basal ganglia—Nucleus accumbens region, Putamen_BG = Basal ganglia—putamen region. Full results for all 53 tissues can be found in S1 and S2 Figs.

Tissue-enrichment of MCP-associated gene expression was analysed using FUMA, which implements a MAGMA gene-property analysis [52] using GTEx [53] gene expression datasets to determine association between trait-associated genes and expression in a range of bodily tissues. MCP-associated genes in females were found to be enriched for expression in the brain, particularly the cerebellum, and frontal cortex (Fig 4). There was no significant tissue enrichment for expression of MCP genes found in males (Fig 4).

**Fig 5:**
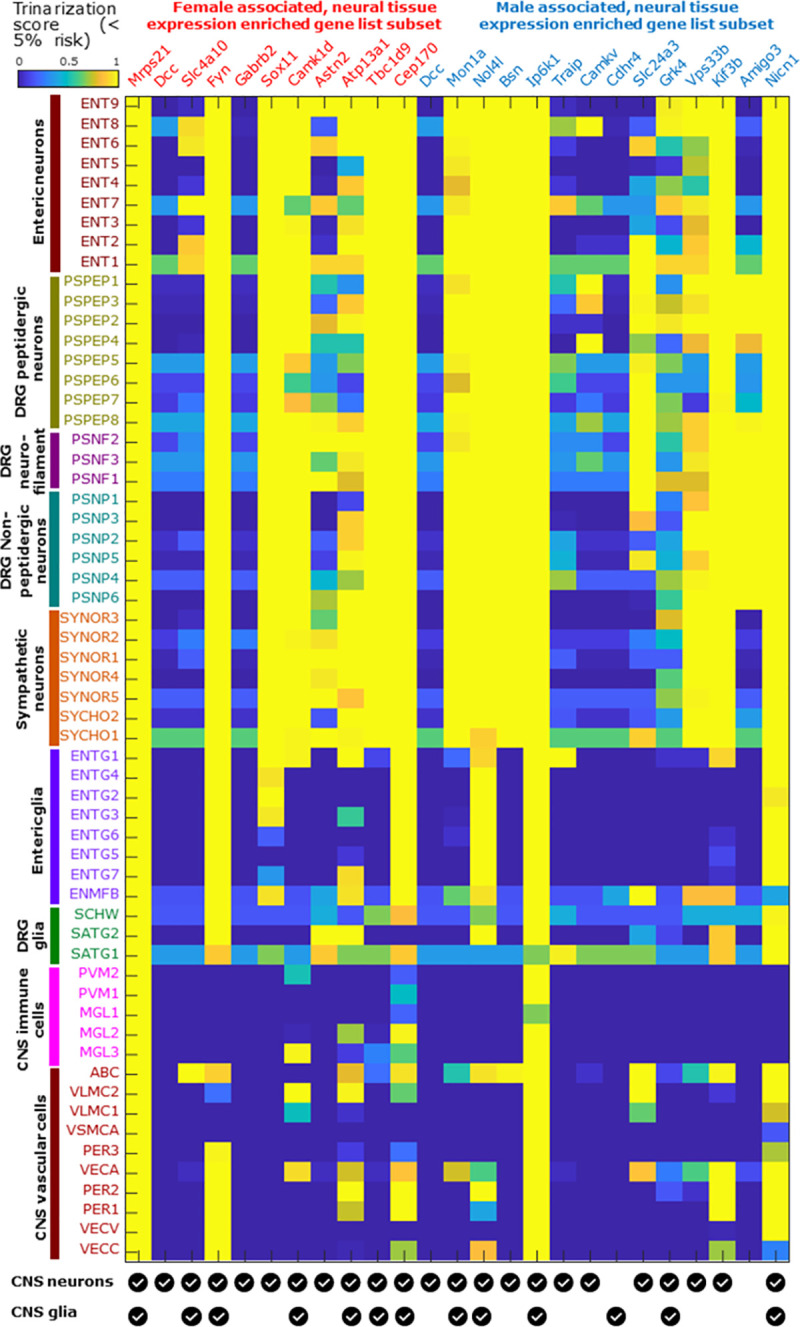
Mouse cell-type-specific expression patterns for sex-differentiated MCP-associated genes. The mousebrain.org database was used to identify cell types of expression in the mouse nervous system (brain neurons and glia have been collapsed to two lines at the base of the plot for clarity) for orthologues of genes identified in this study. Trinarization score (posterior probability of detection in a particular cell type) was used, rather than expression levels. The analysis was carried out for 25 genes selected from the male-specific (n = 14) and female-specific (n = 11; note that *Dcc* appears in both lists) MCP-associated sets as being enriched for neural tissue expression (neural proportion score > 0.5, S4 and S5 Tables). While most of these show pan-neuronal expression, a few are expressed in mouse glial subpopulations (*Cdhr4*), primarily in CNS neurons (*Gabrb2*), primarily in DRG neurons (*Amigo3*), or in limited subsets of neurons (*Dcc*, *Slc4a10*, *Camkv*).

For genes identified (using MAGMA, as reported above) as significantly associated with MCP in either the male-only or female-only GWAS, we carried out further analyses of gene expression at tissue and cell-type level by querying existing transcriptomic datasets, focusing on neural tissues and tissues specific to each sex. For most tissues GTEx data were used, but because GTEx does not contain data for dorsal root ganglion (DRG) neurons, which are key for the generation of the nociceptive signals that initiate pain in chronic pain patients, we assessed expression in this tissue using other comparable datasets [54], which additionally contained measures of gene expression enrichment in human neural tissues (neural proportion score) and in the DRG (DRG enrichment score) (details in Methods). We also used single cell RNA-seq datasets to estimate whether genes of interest are likely to be expressed in neurons in the peripheral or central nervous systems (CNS). Full results of this analysis are given in S4 and S5 Tables, while those for a subset of genes (those enriched for neural tissue expression) are shown in Fig 5. Most of the 37 genes identified from the male-only GWAS were observed to be expressed in the nervous system. Two of the 37 genes, *IP6K3* and *FAM129A*, had low neural proportion scores, suggesting that they are non-neuronal and non-glial. All 37 genes, however, showed DRG expression, although expression level was very low for *DCC* and *IP6K3*, which were more highly expressed in the CNS. One gene, *AMIGO3*, was found to be enriched solely in the DRG; its orthologue has also been found to be primarily expressed only in mouse DRG neurons in the www.mousebrain.org dataset (Fig 5). Of the 30 female-specific MCP-associated genes, all showed high neural expression except *CPS1*, which was not expressed in neural tissue at all. Again, we noted that all 30 genes except *GABRB2* (whose mouse orthologue is primarily expressed in CNS neurons, Fig 5) did show expression in DRG (S5 Table), though none of them showed enriched expression in the DRG compared to the CNS.

Several genes in either the male-only and female-only lists (e.g. *CPS1*, *SEMA3F*, *MST1*, *MST1R*, *SDK1*, *ECM1*; S4 and S5 Tables) that were expressed in neural tissues but with low (< 0.5) neural proportion scores were found to be involved in immune function. Among the genes with high (> 0.5) neural proportion scores, mouse orthologues of many are pan-neuronal in expression based on the mousebrain.org dataset (Fig 5), with a few genes having ubiquitous but neural-enriched expression (*Mrps21*, *Ip6k1*), solely glial expression (*Cdhr4*), or being expressed in limited neuronal subpopulations including CNS, sensory and enteric neuronal subtypes (*Dcc*, *Camkv*, *Slc4a10* respectively). Several of the neuronally expressed genes are known to be involved in axon pathfinding and neurite outgrowth. Many of these genes have elevated expression levels in sex-specific tissues like testis and ovary, and / or are somewhat differentially expressed (10% or more difference in median TPMs across sexes) between male and female cohorts in brain sub-regions in the GTEx database (S4 and S5 Tables), suggesting they may be androgen- or estrogen-regulated.

### Pathway analyses

No significant results were found for male MCP-associated genes using FUMA GENE2FUNC [55]. There was no overrepresentation of male MCP-associated genes within any of the Molecular Signatures Database (MSigDB) gene sets (h, c1-c7) [56–58]. Two of the male MCP-associated genes were associated with disease-related entries in OMIM (Online Mendelian Inheritance in Man) [59], neither of which mention a pain-related phenotype, and none of the genes were listed as previously identified drug targets in DrugBank [60] (S6 Table). Amongst the female MCP-associated genes, overrepresentation was only found for two of the positional gene sets listed in MsigDB (S3 Fig). Nineteen female MCP genes were associated with an OMIM entry, and five were listed as drug targets in DrugBank (S7 Table).

### Polygenic risk score analysis

Polygenic risk scores were used to assess whether MCP and chronic widespread pain (CWP), a related but distinct chronic pain phenotype, are likely to be related biologically. For both men and women, the sex-specific PRS-MCP was significantly associated with CWP (p < 2 x 10^−17^; O.R. = 1.0034 (female) and 1.0026 (male); S8 and S9 Tables), indicating that increased genetic risk for MCP is significantly associated with having chronic widespread pain.

## Discussion

### Comparing male and female multisite chronic pain

Prevalence, coping strategies, and, potentially, mechanisms of development and maintenance of chronic pain vary between the sexes. To explore underlying genetic differences that may contribute to these sex differences in chronic pain, we carried out a large-scale sex-stratified GWAS of a quantitative chronic pain phenotype, MCP. We found both male and female MCP to be moderately heritable. Although the estimated female SNP heritability was higher than that in males (12.5% versus 10.6% respectively), this difference was not significant.

### MCP-associated SNP loci

Twice as many genomic risk loci were identified in GWAS analyses in females as in males (10 versus 5 respectively), with no risk loci shared between the sexes. This may be due to lower sample size in the male MCP GWAS, both in comparison to previous non-stratified analyses and to sample size in the female MCP GWAS analysis (roughly 30,000 more participants are included in the female MCP GWAS than in the male GWAS). Loci were found across the genome (Fig 1 and Table 3) in both males and females, with genomic location varying by sex. We note also, however, that significant loci were found in both men and women on chromosome 6, only 0.89 Mbp apart (Table 3).

Additional trait-associated SNPs were discovered when the sex-stratified GWAS outputs were meta-analysed, likely due to increased power. Twenty-four loci that had not reached genome-wide significance in our previous sex-combined analysis were found to be associated with MCP after meta-analysis. The fact that these loci were not identified in the non-stratified GWAS could be due to effect heterogeneity, in terms either of direction or magnitude, between the sexes reducing the overall signal at these loci.

Previous studies have highlighted sex-specific or sex-differentiated loci in a range of disorders and traits, such as ASD, anthropometric traits and asthma [31,61–63]. Four of the loci found to be associated with MCP in our meta-analysis of sex-stratified GWAS outputs, including 7 SNPs in total, showed signs of heterogeneity of effect size between the sexes (I^2^ p < 0.05), though the evidence was not significant after FDR-adjustment. Nevertheless, if these results are replicated in future studies, it may be that these loci are found to contribute to sex differences in chronic pain.

### Genes of interest

#### Genes associated with MCP in males

The sex-stratified gene-level analysis discovered a number of genes with significant evidence for a genetic contribution to MCP. Significant genes in males included *CENPW*, *MTCH2*, *NICN1*, *AMIGO3*, *DNAJA4*, *CTBP2* and *NOP14*, with the latter two also being significant in the meta-analysis gene-level testing (S2 and S3 Tables). *CENPW* encodes centromere protein W, involved in kinetochore assembly and function, and associated with diseases such as type 1 diabetes [64–66]. *MTCH2* (mitochondrial carrier 2) encodes a member of the SLC25 family, a family of transporters localised to the inner membrane of mitochondria and involved in a wide range of cell metabolism functions [67]. SNPs in this locus have previously been associated with obesity [68–70], and this gene may be involved in regulation of development of adipocytes. *NICN1* (nicolin 1) encodes a nuclear protein of unknown function expressed in a variety of tissues [71]. *AMIGO3* (adhesion molecule with Ig-like domain 3), the only male-specific MCP-associated gene whose expression was enriched in DRG, is a member of a small family of cell-surface immunoglobulin domain- and leucine-rich repeat-containing adhesion molecules. Its function is not well understood, but it is expressed in a range of DRG neuronal subtypes and may play a specialized role in nociception or other sensory modalities. Interestingly, *AMIGO3* is located almost next to and within 40 kbp of *IP6K1*, which was also found to be associated in the male gene-level analysis (S2 Table). It may be that there is coordinate regulation of these two genes in tissues relevant to MCP, or that a number of separate functional variants are distributed across this genomic locus but in fact only influence expression of one of these two genes. *DNAJA4* (DnaJ Heat Shock Protein Family (Hsp40) Member A4) encodes a heat shock protein [72] previously shown to be involved in melanoma metastasis and angiogenesis regulation, but is generally poorly characterised [73].

#### Genes associated with MCP in females

Genes found to be associated with MCP in females included *NCAN*, *SPATS2L*, *TBC1D9*, *CAMK1D*, *SOX11*, *GON4L*, and *DAGLB*, the last of which was also significantly associated in the meta-analysis. *NCAN* (neurocan) encodes a chondroitin sulfate proteoglycan [74] potentially involved in the modulation of cell adhesion and migration, and previously linked to bipolar disorder in GWAS and mouse model studies [75,76]. *SPATS2L* (spermatogenesis associated serine rich 2 like) encodes a protein that may be involved in ribosome biogenesis and translational control as a response to oxidative cellular stress [77]. *TBC1D9* (TBC1 domain family member 9) encodes a potential GTPase and was found to be overexpressed in mantle cell lymphoma [78]. *TBC1D9* was also recently found to be involved in a Ca^2+^-dependent cellular response to infection [79]. *CAMK1D* (calcium/calmodulin dependent protein kinase ID) encodes a member of the calcium/calmodulin-dependent protein kinase 1 family involved in granulocyte regulation, activating CREB-dependent gene transcription, the activation and differentiation of neutrophils, promotion of basal dendritic growth of hippocampal neurons, and apoptosis in erythroleukemia cells [80]. *SOX11* (SRY-box transcription factor 11) encodes a member of the SOX (SRY-related HMG-box) family of transcription factors, with potential roles both in nervous system development and in neurogenesis during adulthood [81–85]. *De novo* mutations in this gene have also been associated with Coffin-Siris syndrome [86]. *GON4L* (Gon-4 like) encodes a protein involved in transcriptional repression [87,88]. *DAGLB* (diacylglycerol lipase beta) encodes an enzyme that participates in the endocannabinoid synthesis pathway and is required for axonal growth during development and for retrograde synaptic signalling in mature synapses [89].

Overall, it was notable that genes found to be significantly associated with male and female MCP were largely different. Only one gene, *DCC* (DCC netrin 1 receptor; a.k.a. deleted in colorectal carcinoma), was associated with both male and female MCP. *DCC* encodes a receptor for the guidance cue netrin 1, and is important for development of the nervous system, particularly the dopaminergic system [90]. Mutations in the *DCC* gene have been found in those with congenital mirror movement disorder (MRMV-1; [91] and it has also been previously associated with a range of complex brain-related traits, including suicidality, mood instability, intelligence and putamen volume [41,42,92–94]. *DCC* has also been highlighted as a risk gene for major depression, and may be involved in the pathology of depression through effects on axon guidance in the developing and adult CNS [95,96].

#### Gene expression differences in male and female MCP

Gene expression analyses in GTEx (which does not have DRG samples) carried out using FUMA indicated that expression of the female-specific MCP-associated genes was enriched primarily in brain tissue, and this pattern was also seen when meta-analysed sex-stratified GWAS outputs were analysed similarly (not shown). Almost all of these genes were also expressed in the human DRG—it is interesting to speculate as to whether the role of these genes in initiation or maintenance of chronic pain phenotypes is mediated through roles in the brain or via effects on cells located within the DRG. No significant enrichment for specific tissues was seen in analyses of male MCP-associated genes using GTEx and the human DRG expression profiles. The lack of tissue-enrichment findings for the male-specific genes may, as with the lower number of MCP-associated loci, be due to reduced power resulting from lower sample size in the male GWAS. However, these patterns of tissue-level gene expression in GTEx may also indicate differing gene expression between the sexes, with more ubiquitous expression across all tissues for genes associated with MCP in males, while genes conferring risk in females may tend to have more tissue-specific expression patterns. These expression patterns may also be associated with the fact that the GTEx resource is enriched for male tissue samples (V8 release; 67.1% male)–sex-differential enrichment of certain genes may be conflated with tissue-differential gene enrichment.

It is still notable that almost all the male or female MCP-associated genes were found to be expressed in human CNS tissues and in DRG. A subset of these are enriched in human neural tissues and additionally are expressed in mouse neuronal subpopulations when examining single cell sequencing databases (Fig 4). All of these lines of evidence, together, suggest putative central and peripheral neuronal roles for some of these genes, many of which have not been historically well studied in the field of chronic pain.

#### Genetic correlations

For both males and females, MCP was genetically correlated with non-stratified MCP [17], at r_g_ = 1. In contrast, the genetic correlation between male MCP and female MCP was found to be high but significantly less than 1 (r_g_ = 0.92), with around 8% of common SNP-tagged trait variation therefore not shared between the two traits. However, it could be argued that although this figure suggests that a small subset of genetic variation linked to MCP is unique to each sex, this difference is not large enough to consider MCP in the two sexes as biologically distinct to a considerable extent—traits correlated at lower r_g_ are routinely used as proxies for one another in GWAS settings e.g. educational attainment as proxy for intelligence (r_g_ ~ 70%) [92], or current age as a proxy for life span (r_g_ ~40–70%) [97].

Genetic correlations with a range of psychiatric disorders, psychological traits and somatic traits and disorders were explored and notable sex-related commonalities and differences were observed. Significant genetic correlations of similar magnitude for both sexes were found between MCP and a range of psychiatric, autoimmune and anthropometric traits. Some phenotypes were significantly genetically correlated only with female MCP (schizophrenia, r_g_ = 0.13; PGC cross-disorder phenotype, r_g_ = 0.14; PTSD, r_g_ = 0.44; anorexia nervosa, r_g_ = -0.08), while others were significantly genetically correlated only with male MCP (autism spectrum disorder, r_g_ = -0.16; primary biliary cholangitis, r_g_ = 0.14). In most cases, where one sex was significant and the other was not, the r_g_ values for both sexes were not very different, suggesting that the underlying biology is only subtly, quantitatively different. The sex differences that were observed may reflect sex-differential patterns of pleiotropy, with some genetic factors contributing differentially to multiple phenotypes in males and females. It would be of interest to investigate the relative effect size of loci contributing to MCP and to schizophrenia and PTSD in the two sexes and the biological mechanisms underlying this difference.

Observed genetic correlation differences may also, however, be due to differences in sample size between male and female MCP GWASs (with lower sample size for men compared to women). Differences may also be a result of either men or women being over-represented in the comparison GWAS. For example, in the GWAS meta-analysis of autism spectrum disorder [98] contributing cohorts had M:F ratios from 1.2:1 to as high as 8.6:1, PBC GWASs contributing to the discovery set in the PBC GWAS meta-analysis [99] contained > 90% female cases, and the anorexia nervosa GWAS [100] contained only female cases. Analysis of the cohorts used in the schizophrenia and PTSD GWAS meta-analyses [101–106], suggests that the sex difference in genetic correlation with MCP for these phenotypes is not primarily driven by an overrepresentation of one sex.

### Comparing the relationship between chronic widespread pain and MCP in males and females

Each sex-specific PRS was significantly associated with CWP in the corresponding sex, but the magnitude of association was much lower in comparison to the sex-combined PRS analysis reported previously [17], which may reflect the smaller sample sizes in the sex-stratified analyses. As previously found in sex combined analyses, results indicated a moderate degree of shared genetic basis for MCP and chronic widespread pain in both sexes, with degree of sharing potentially slightly stronger in females than in males. It is possible that this difference is driven at least in part by the overrepresentation of females in CWP cases (M:F ratio 1:1.74).

### Clinical perspective on findings and potential impact on treatment

Overall, our findings suggest that MCP shows genetic (and therefore biological) differences between men and women. If women with MCP are more likely to be at risk of PTSD and schizophrenia (and *vice versa*) specific screening for these potential comorbidities could be appropriate, with a view to instigating additional appropriate management or referrals. Except for PTSD, however, these sex-differential genetic correlations were relatively small in comparison with those that affected both sexes, particularly a range of mood phenotypes. Nevertheless, the shared biology evident in the significant genetic correlations should stimulate investigation of whether screening for these potential clinically important co-morbidities can be used to improve management of chronic pain patients.

Enhanced attention to the sex differences in manifestation and underlying biology of chronic pain is also merited, and may lead to improvements in clinical assessment, awareness of risks and choice of medical treatment. For example, if MCP in women is more strongly associated with immune function (based on evidence from MCP-associated genes), efficacy and side-effect profiles for drugs targeting immune system function may be different in women and men. Inappropriate treatments, such as chronic opioid prescribing, might also have sex-differential consequences—opioids are known to adversely affect immune function [107,108]. These sex differences in MCP biology may also inform the search for new or re-purposed drugs that can be prescribed in a sex specific manner. It is already known that specific proteins play sex-specific roles in pain processing and that certain drugs have been found to have sex-specific analgesic effects (reviewed by [109]).

### Limitations

These analyses were carried out using UK Biobank, which was used in our previous sex-combined MCP GWAS [17]. In comparison to our previous analysis, sample size in each individual sex-stratified GWAS was lower, which leads to somewhat reduced power. However, sample sizes are still larger than many sex-combined GWAS analyses of chronic pain phenotypes, and meta-analysis of the sex-stratified GWAS outputs resulted in an increase in power to find MCP-associated SNPs overall. True replication is difficult due to heterogeneity in chronic pain phenotyping and available sample sizes of potential independent cohorts, but in an independent subset of the UK Biobank, a PRS constructed from each sex-stratified GWAS output was found to be significantly associated with CWP, a related but distinct chronic pain phenotype of interest.

Although this work was focused on sex differences in the genetics of MCP we examined only autosomal variation. An important extension of this work would be to assess genetic associations with X chromosome loci, which is likely to provide an additional heritability contribution and give a fuller picture of sex differences in MCP at the genetic level. Inclusion of the X chromosome in GWAS analyses is associated with specific methodological and statistical issues including lower quality genotyping array coverage of the X chromosome compared to autosomes, differences in how imputation needs to be implemented, differences in X chromosome dosage between the sexes leading to differences in population genetics/demographic history of the X chromosome relative to the autosomes, and changes to quality control required (and differing QC protocols between sexes) [110,111]. We aim to address this in future work as we adapt our BOLT-LMM pipeline and downstream analysis pathway.

These GWAS analyses were carried out on a white British subset of UK Biobank, and therefore may not generalise to admixed or non-white populations. GTEx donors are also primarily white (v8 release: 84.6% white, 12.9% African American, 0.2% American Indian, 1.3% Asian, 1.1% Unknown, [https://www.gtexportal.org/home/tissueSummaryPage]), as are donors who provided DRG tissue (white females [54]), again potentially limiting generalisability of our findings to non-white populations.

## Conclusions

Sex differences in chronic pain likely have, at least in part, a genetic basis and the study of complex traits such as chronic pain is likely to benefit from “sex-aware” analytical approaches. This study comprises one of the largest sex-stratified genetic analyses of a chronic pain phenotype, and highlights sex-differential MCP-associated loci, genes, genetic correlations, and patterns of tissue expression. We also examined transcriptome abundance of key sex-differential MCP-associated genes in a range of neural and non-neural tissues, including DRG, an important nervous system component in chronic pain which is not part of the GTEx resource and so may be understudied in GWASs with follow-up conducted solely using FUMA.

Sex-stratified GWASs can provide an increase in power if heterogeneity in effects of trait-associated variants is seen between the sexes. Here, 24 novel genes and 11 novel independent lead SNPs were associated with MCP, in addition to the findings from previous non-sex-stratified work, further contributing to understanding of genetic variation predisposing to chronic pain.

Genetic correlation results indicated possible sex-differential pleiotropy, including differing genetic correlations between certain psychiatric disorders and traits and chronic pain in women compared to men. However, it is of note that genetic correlations between the sexes are largely similar, particularly with respect to psychiatric disorders and traits including MDD, anhedonia and depressive symptoms. This is the first study to use novel GWAS outputs from studies of suicidality and of RDoC mental health traits such as mood instability and anhedonia in genetic correlation analyses with chronic pain and it provides an important insight into shared genetic factors between these comorbidities of chronic pain and MCP.

The patterns of gene expression enrichment associated with the identified predisposing genes supports and enhances our previous conclusion that MCP derives more strongly from brain and/or CNS-based mechanisms than from other organ systems and functions. We did observe, however, that almost all the sex-specific MCP-associated genes identified are expressed in the DRG as well as in the brain, with one sex differentiated MCP gene, *AMIGO3*, being DRG-specific amongst the tissues assessed, raising the possibility that cells in the DRG that play a specialised role in nociception or other sensory function might be involved in chronic pain mechanisms. We also observed that several MCP-associated loci in both sexes have been linked to immune function.

Overall, our findings indicate the existence of potential sex differences in chronic pain at multiple levels, from SNP-level to transcript abundance and the results support theories of strong nervous system and immune involvement in chronic pain in both sexes. These findings may inform development of novel treatment approaches in future, as well as adding to our understanding of the physiology of chronic pain.

## Methods

### Chronic pain phenotyping

UK Biobank participants were asked via a touchscreen questionnaire about “pain type(s) experienced in the last month” (field ID 6159), with possible answers: ‘None of the above’; ‘Prefer not to answer’; pain at seven different body sites (head, face, neck/shoulder, back, stomach/abdomen, hip, knee); or ‘all over the body’. The seven individual body-site pain options were not mutually exclusive, but those who chose ‘all over the body’ could not also select from the seven individual body sites. Where patients reported recent pain at one or more body sites, or all over the body, they were additionally asked (category ID 100048) whether this pain had lasted for 3 months or longer.

Chronic Widespread Pain (CWP) was defined as reported [112], and the ‘case’ group included only those participants who answered that they had pain ‘all over the body’ that was longer than 3 months in duration in the touchscreen questionnaire. These individuals were excluded from analyses of other chronic pain phenotypes as there is some evidence that this phenotype can be substantially different from more localised chronic pain [113]. Multisite Chronic Pain (MCP) was a quasi-quantitative variable defined as previously reported [17]; briefly, this variable captures the number of body sites at which chronic pain (at least 3 months duration) was recorded (excluding those with CWP): phenotypic values therefore ranged from 0 to 7. 10,000 randomly selected individuals reporting no chronic pain were excluded from the GWAS to use as controls in subsequent polygenic risk score (PRS) analyses.

### Genetic quality control

For the GWAS analyses, SNPs with an imputation quality score of less than 0.3, minor allele frequency (MAF) < 0.01 and/or Hardy-Weinberg equilibrium (HWE) test p < 10^−6^ were excluded. Participants whose self-reported sex did not match their genetically-determined sex, those who had putative sex-chromosome aneuploidy, those considered outliers in UK Biobank QC in terms of missingness or heterozygosity [114], and those who were not of self-reported white British ancestry were excluded from analyses. A list of “poor quality” samples (due to missingness, putative genetic and reported sex mismatch and/or unexpectedly high heterozygosity) was derived by Bycroft et al [114] and is available to all researchers using UK Biobank, and was used here as part of genetic quality control. Briefly, putative sex chromosome aneuploidy was defined by visual inspection of scatterplots of mean log2 ratio (L2R) on X and Y chromosomes, and 652 UKB participants meet these criteria for putative sex-chromosome aneuploidy (Supplemental Information S 3.6 [114]). Samples with a heterozygosity value, adjusted for both ancestry and genetic principal components (GPCs), above the mean heterozygosity value (0.1903) and missing rate greater than 0.05 as computed using PLINK ‘—miss’ command were also flagged as potentially poor quality ([114]; 968 such samples are listed in this paper’s Supplemental Information S 3.5.3).

A summary of participant MCP phenotypic information for those included in each GWAS is shown in Table 4.

**Table 4:** Number of participants per MCP phenotype level group included in each GWAS (male or female sex-stratified analysis).

MCP	N female	% female	N male	% male
0	113148	54.11	105474	59.07
1	49984	23.91	42734	23.93
2	26000	12.43	18612	10.42
3	12376	5.92	7771	4.35
4	5319	2.54	2970	1.66
5	1723	0.82	780	0.44
6	471	0.23	181	0.10
7	72	0.03	34	0.02
total n in each GWAS	209093	NA	178556	NA

### BOLT-LMM GWAS and gene-level analysis in FUMA

Sex-stratified GWASs of MCP, modelled as a quantitative trait, were carried out using BOLT-LMM [115], adjusting for age and chip (genotyping array), under the infinitesimal model of genetic risk, as previously described for our unstratified MCP GWAS [17]. BOLT-LMM uses a genetic relatedness matrix in the model to adjust for population stratification and familial relationships. We therefore did not include genetic principal components (GPCs) in the model. The SNP-level GWAS summary statistics were then analysed using FUMA [55] to obtain genome-wide plots and carry out MAGMA [49] gene-set and gene-based test analyses and gene expression analysis using GTEx [53] for male-enriched and female-enriched MCP genes. Significant independent lead SNPs were determined according to FUMA. Briefly, FUMA defines lead SNPs as the subset of independent significant SNPs (SNPs associated with the trait at p < 5 x 10^−8^ and having LD r^2^ < 0.6 with any other significant SNP) that are not in LD (r^2^ > 0.1) with any other lead SNP [55]. In addition, when these LD blocks of independent significant SNPs are in close proximity (< 250kbp apart), separate loci based on LD thresholds are merged into a single genomic locus, and thus each genomic risk locus can contain multiple lead and independent significant SNPs [55].

#### Meta-analysis of male and female GWAS Summary statistics

Meta-analysis of the two sex-specific GWAS summary statistics datasets was carried out using METAL [116], deploying a fixed-effects model and weighted by standard error (‘SCHEME STDERR’) with default options aside from selecting ‘heterogeneity’ in order to analyse heterogeneity (‘ANALYZE HETEROGENEITY’). A meta-analysis p-value of < 5 x 10^−8^ was selected as the significance threshold for association. Gene-level analysis (MAGMA) was also carried out using meta-analysis output.

### Transcriptome analysis of sex-specific association gene lists

We further analysed the tissue and cell type of expression of male-specific and female-specific genes identified from the MAGMA gene-based analysis of the sex-stratified GWAS results. Specifically, we characterized gene expression in mammalian nervous system tissues and cell types as a potential starting point for identifying the functions of these genes with respect to pain. We also characterised expression for the one gene found to be associated with both male and female MCP in the gene-based analyses (*DCC*).

RNA-seq-derived gene expression values have been previously reported [54] as relative abundances in TPMs (standardised Transcripts per Million mapped reads) for 12 adult human tissues (6 neural and 6 non-neural). Additionally, the study notes 3 metrics on a scale of 0 to 1 for each gene based on expression in these 12 tissues: normalized Shannon’s entropy as a measure of tissue specificity (0 for highly tissue-specific and 1 for tissue-agnostic gene expression in the quantified tissues), neural proportion score as a measure of enriched expression (possibly neuronal and/or glial) in the nervous system (0 for genes not expressed in the nervous system and 1 for genes expressed solely in neural tissues with respect to these 12 tissues), and DRG enrichment score for identifying specificity of gene expression in the DRG with respect to the other 11 profiled tissues (0 for no expression in the DRG or for tissue-agnostic gene expression, and 1 for DRG-specific gene expression in the context of the set of profiled tissues). The mathematical formulations for these scores are provided in detail in Ray *et al*. 2018 [54]. The corresponding tables are presented in S4 Table (male) and S5 Table (female).

Genes with neural proportion scores > 0.5 (with overall more neural than non-neural tissue expression), were further characterized by the putative cell type(s) of gene expression in the mammalian nervous system (Fig 4). While human single cell resolution RNA-seq datasets are not publicly available for the peripheral nervous system, a comprehensive database of gene expression in the mouse nervous system exists: the www.mousebrain.org repository [117]. While it is true that nervous system expression patterns may be different between human and mouse between nervous system subpopulations due to regulatory evolution or due to differences in nervous system cell types [118], it is unlikely that expression would change categories across the 4 broad nervous system cell type categories: neurons, glia, immune and vascular cells) between human and mouse, given overall conservation of tissue gene expression profiles across humans and mice [54]. Trinarization scores, defined as the posterior probability (using a Bayesian framework) of detecting reads from a particular gene in a cell type subpopulation (details are given in ref. [117]), were used to characterise gene expression by cell-type. Fig 4 visualizes trinarization scores for a range of peripheral nervous system cell types (sensory neurons and glia, enteric neurons and glia, and sympathetic neurons). While peripheral nervous system vascular and immune cells have not been profiled so far in www.mousebrain.org, we depict CNS vascular immune cells and vascular cells as surrogate cell types for their PNS counterparts. Both CNS neurons and glia play a critical role in chronic pain, but due to high diversity of cell types were not ideal for summarizing expression patterns in each subpopulation of these categories succinctly in a single figure. Instead, summary rows for CNS neurons and glia expression for relevant genes are provided at the bottom of the figure. For further details of expression in these subtypes, www.mousebrain.org can be queried for mouse gene expression profiles.

Finally, for genes with neural proportion scores > 0.5, the GTEx database was queried for sex differential gene expression in profiled CNS regions, and for high expression in sex-specific tissues, such as testis and ovary, noted in the comments section of S4 and S5 Tables.

### Genetic correlations

Genetic correlations with a range of neuropsychiatric disorders and traits were assessed by LD-score regression (LDSR) for the male and female MCP GWAS outputs separately. Summary statistics datasets employed were publicly available or available via LD Hub [119,120], or were results from published and unpublished in-house GWASs. Pre-computed LD scores were used, along with HapMap3 SNPs as a reference. By default the munging part of LDSR filters out SNPs with an info score of < 0.9, a MAF of < 0.01, a GWAS p value outside the range 0–1, strand-ambiguous SNPs, non-SNP (e.g. indel) variants, and SNPs with low sample size. The default option of no constraint on the LDSR intercept was used. LDSR p-values for genetic correlation were FDR-corrected within each sex. LDSR was also used to assess trait polygenicity and to calculate a SNP-heritability estimate. Note that although some of the GWAS results used in this analysis came from studies that included UK Biobank data, genetic correlations estimated using LDSR are not subject to bias caused by sample overlap [121].

### Polygenic risk score analysis

Previous analyses showed that a polygenic risk score (PRS) for MCP was associated with the phenotype of chronic widespread pain (CWP) in women but not in men, and that this PRS was associated more strongly with chronic pain phenotypes in women than in men in an independent cohort (Johnston K.J.A. *et al*., unpublished). To further explore the relationship between MCP and CWP, we assessed how separate male and female PRSs for MCP were associated with CWP. Separate sex-specific PRSs for MCP, based on the sex-specific GWAS results reported here, were calculated for a ‘case’ group consisting of participants who reported pain all over the body that lasted for three months or longer (a proxy phenotype for Chronic Widespread Pain; CWP; N = 6, 813)), and for a ‘control’ group consisting of 10,000 randomly selected UKB controls, both of which had been excluded from the GWAS analyses (demographic data for this subsample are given in Table 5).

**Table 5:** Summary of sample sizes of participants used in each of the two PRS analyses (male or female).

	Female		Male		Combined Totals	
	N	mean age (years)	N	mean age	N	mean age
Control	5135	56.68	4865	56.96	10000	56.81
CWP	4328	57.00	2485	57.27	6813	57.10
Total	9463	56.83	7350	57.07	16813	56.93

SNPs associated with MCP at p < 0.01 in the original sex-specific GWAS were selected and LD-pruned (at a threshold of r^2^ < 0.1 within a 250kbp window using the PLINK ‘—clump’ command). Sex-specific PRS-MCPs were calculated for each individual in the analysis as the sum of risk alleles at each SNP, weighted by effect size (beta value) in the GWAS [122]. PRS values were standardised and z-scores were used in the analysis. Association between standardised sex-specific PRS-MCP and CWP status was investigated separately in males and in females in the target case-control subsample using logistic regression, adjusted for chip (genotyping array), age and the first eight genetic principal components.

## Supporting information

S1 Table Genetic correlations between MCP and other disorders and traits. Results of LDSR analysis using summary statistics from the sex-stratified GWASs of MCP versus a range of potentially related disorders and traits. Genetic correlations are given as r_g_ values (and FDR-corrected p-values) sorted in order of numerically decreasing r_g_ for female MCP vs other traits. f_rg and m_rg = genetic correlation value for female and male MCP versus trait, respectively, f_p_fdr and m_p_fdr = FDR-corrected p value for genetic correlation, source = source of trait GWAS data, PMID = PubMed ID of associated publication for GWAS of trait. Significant genetic correlations (FDR-corrected p value < 0.05) within each sex are highlighted orange, non-significant in blue.
(PDF)

S2 Table Table accompanying Venn diagram of genes associated with multisite chronic pain in MAGMA gene-level analyses. ‘Found in’ refers to the GWAS and corresponding MAGMA gene-level analyses where genes were found to be significantly associated with MCP: Female = sex-stratified GWAS (female), Male = sex-stratified GWAS (male), Meta = GWAS meta-analysis of male and female sex-stratified GWAS outputs, Original = sex-combined GWAS analysis described previously [17]. Total = total number of genes in category. Elements = gene names.
(PDF)

S3 Table Genes associated with MCP in MAGMA gene-level analyses of GWAS meta-analysis output. (PDF)

S4 Table. Expression of genes associated with male MCP in MAGMA analyses across neural and non-neural tissues. TPM = transcripts per million, DRG = dorsal root ganglion. h_DRG_enrich = DRG enrichment score. h_entropy = normalized Shannon’s entropy. h_neural_propn = neural proportion score (PDF)

S5 Table Expression of genes associated with female MCP in MAGMA analyses across neural and non-neural tissues. TPM = transcripts per million, DRG = dorsal root ganglion. h_DRG_enrich = DRG enrichment score. h_entropy = normalized Shannon’s entropy. h_neural_propn = neural proportion score.
(PDF)

S6 Table OMIM and DrugBank derived information (male MCP). (PDF)

S7 Table OMIM and DrugBank derived information (female MCP). (PDF)

S8 Table Association between male MCP PRS and CWP in men. *Full results (chip, PCs) not shown for brevity. SE = standard error, Z = Z value, P = p value, OR = odds ratio, PRS = z-standardised PRS value.
(PDF)

S9 Table Association between female MCP PRS and CWP in women. *Full results (chip, PCs) not shown for brevity. SE = standard error, Z = Z value, P = p value, OR = odds ratio, PRS = z-standardised PRS value.
(PDF)

S1 Fig Tissue Expression of MCP-associated Genes (male) (53 tissues). (PDF)

S2 Fig Tissue Expression of MCP-associated Genes (female) (53 tissues). (PDF)

S3 Fig FUMA gene set analysis results for female MCP genes from the gene-level analysis. (TIFF)
