# Supplementary material for: Sex-stratified genome-wide association study of multisite chronic pain in UK Biobank
Source: PLoS Genet. 2021 Apr 8;17(4):e1009428. doi: 10.1371/journal.pgen.1009428 (PMC8031124; doi:10.1371/journal.pgen.1009428)
Supplement: S1 Table — Results of LDSR analysis using summary statistics from the sex-stratified GWASs of MCP versus a range of potentially related disorders and traits. Genetic correlations are given as rg values (and FDR-corrected p-values) sorted in order of numerically decreasing rg for female MCP vs other traits. f_rg and m_rg = genetic correlation value for female and male MCP versus trait, respectively, f_p_fdr and m_p_fdr = FDR-corrected p value for genetic correlation, source = source of trait GWAS data, PMID = PubMed ID of associated publication for GWAS of trait. Significant genetic correlations (FDR-corrected p value < 0.05) within each sex are highlighted orange, non-significant in blue. (PDF) [file pgen.1009428.s001.pdf]

| Trait | f_rg | f_p_fdr | m_rg | m_p_fdr | source | PMID |
|---|---|---|---|---|---|---|
| Anhedonia | 0.62 | 2.1E-103 | 0.69 | 4.6E-71 | In-house analysis | 31797917 |
| Depressive symptoms | 0.59 | 1.4E-51 | 0.61 | 5.4E-32 | ld_hub | 27089181 |
| MDD | 0.54 | 5.8E-56 | 0.55 | 1.3E-51 | PGC | 29700475 |
| Relative Amplitude | 0.51 | 0.09 | 0.44 | 0.11 | In-house analysis | 30120083 |
| Mood Instability | 0.48 | 3.9E-75 | 0.54 | 1.7E-37 | In-house analysis | 31168069 |
| Anxiety (case-control) | 0.46 | <1.00E-120 | 0.44 | 0.002 | PGC | 26754954 |
| PTSD (European Ancestry) | 0.44 | 0.002 | 0.30 | 0.06 | PGC | 28439101 |
| Suicidality | 0.44 | 6.1E-28 | 0.34 | 2.1E-16 | In-house analysis | 30745170 |
| Neuroticism | 0.40 | 7.2E-30 | 0.40 | 2.2E-10 | ld_hub | 27089181 |
| Self Harm | 0.37 | 9.7E-13 | 0.23 | 4.4E-06 | In-house analysis | 30745170 |
| Suicide & Self Harm | 0.36 | 1.0E-10 | 0.37 | 1.0E-08 | In-house analysis | 30745170 |
| BMI | 0.29 | 3.3E-31 | 0.31 | 4.1E-31 | GIANT consortium | 25673413 |
| Asthma | 0.24 | 0.001 | 0.19 | 0.025 | ld_hub | 17611496 |
| PGC cross-disorder analysis | 0.14 | 0.001 | 0.08 | 0.08 | ld_hub | 23453885 |
| Schizophrenia | 0.13 | 2.0E-04 | 0.07 | 0.08 | PGC | 25056061 |
| WHR (BMI-adjusted) | 0.11 | 9.0E-04 | 0.10 | 0.011 | GIANT consortium | 25673412 |
| Systemic lupus erythematosus | 0.08 | 0.14 | 0.03 | 0.72 | ld_hub | 26502338 |
| Primary biliary cirrhosis | 0.07 | 0.22 | 0.14 | 0.04 | ld_hub | 26394269 |
| Bipolar Disorder | 0.06 | 0.21 | 0.01 | 0.75 | ld_hub | 21926972 |
| Rheumatoid arthritis | 0.04 | 0.32 | 0.02 | 0.75 | ld_hub | 24390342 |
| Inflammatory Bowel Disease | 0.02 | 0.60 | 0.04 | 0.62 | ld_hub | 26192919 |
| Crohn's Disease | 0.01 | 0.75 | 0.02 | 0.75 | ld_hub | 26192919 |
| Ulcerative Colitis | 0.01 | 0.78 | 0.03 | 0.75 | ld_hub | 26192919 |
| Celiac disease | -0.04 | 0.52 | -0.12 | 0.09 | ld_hub | 20190752 |
| Parkinson’s disease | -0.04 | 0.45 | 0.04 | 0.47 | ld_hub | 19915575 |
| Autism spectrum disorder | -0.07 | 0.19 | -0.16 | 0.01 | ld_hub | 28540026 |
| Anorexia Nervosa | -0.08 | 2.5E-02 | -0.04 | 0.42 | ld_hub | 24514567 |
| Subjective well being | -0.37 | 7.0E-18 | -0.32 | 7.5E-09 | ld_hub | 27089181 |
