# Supplementary material for: Sex-stratified genome-wide association study of multisite chronic pain in UK Biobank
Source: PLoS Genet. 2021 Apr 8;17(4):e1009428. doi: 10.1371/journal.pgen.1009428 (PMC8031124; doi:10.1371/journal.pgen.1009428)
Supplement: S2 Table — ‘Found in’ refers to the GWAS and corresponding MAGMA gene-level analyses where genes were found to be significantly associated with MCP: Female = sex-stratified GWAS (female), Male = sex-stratified GWAS (male), Meta = GWAS meta-analysis of male and female sex-stratified GWAS outputs, Original = sex-combined GWAS analysis described previously [17]. Total = total number of genes in category. Elements = gene names. (PDF) [file pgen.1009428.s002.pdf]

Found In:	Total	Elements
Female Male Meta Original	1	DCC
Female Meta Original	20	STAG1 TARS2 UHRF1BP1 ATP13A1 FYN ASTN2 ILF3 C6orf106 SNRPC MRPS21 CPS1 MAML3 GABRB2 SLC44A2 RPRD2 ECM1 PRPF3 MLLT10 CEP170 SDK1
Male Meta Original	29	MST1R MON1A SLC25A13 FOXP2 AMIGO3 MST1 RNF123 CAMKV FAM129A FAM120A GMPPB RBM5 BSN RHOA ASXL1 TCTA KIF3B UQCC2 SEMA3F CDHR4 VPS33B C20orf112 BBX GRK4 RBM6 IP6K3 IP6K1 SLC24A3 TRAIP
Female Original	3	NPM1 C1orf51 SLC4A10
Female Meta	1	DAGLB
Male Original	1	UBA7
Male Meta	2	CTBP2 NOP14
Meta Original	70	DNAJC6 ERBB4 ZNF821 PTK2 KIF26B HEXIM2 DNM1 BAI2 RBFOX1 GNAT1 LAMA2 ROBO2 MSL2 PABPC4 GRM3 ASXL3 NUP43 GINM1 CCDC36 LIN28B FHL5 KATNA1 PPP1R13B SMARCC1 PURG EFNB2 AGO2 NRXN1 PHF2 SDCCAG8 VAMP5 NUP210L DAG1 RP11-3B7.1 DHX30 NCAM1 CTD-2330K9.3 FAM172A ZRANB1 SP4 DCAKD TENM2 TM9SF4 MARVELD3 C3orf84 EXD3 LEMD2 TSKU RABGAP1L TCF20 NEBL FAM212B NMT1 PCCB IST1 TCF4 ATXN1L CKAP5 JAKMIP3 NUMB LANCL1 SPHKAP EIF4E3 ZNF101 SCAI PSMD2 PCMT1 PTPRO ERBB3 TRMT13
Female	6	NCAN SPATS2L TBC1D9 CAMK1D SOX11 GON4L
Male	4	CENPW MTCH2 NICN1 DNAJA4
Original	19	SORT1 LATS1 PXT1 ZFYVE21 LAMB2 GATAD2B ZBTB46 CCDC71 RFTN2 PSMA5 F2 AC011997.1 OLFM4 PTBP1 RERG PACSIN1 BOLL GOLGA1 CRTC2
Meta	24	USP4 TEX29 DIAPH3 GNAQ FAF1 AP1G1 C7orf50 PHLPP2 GPER1 RHOT2 SPATS2 SUOX BPTF CRTAC1 LRRC39 NOVA1 TAT PPCDC TMEM132B ITPR3 SKIDA1 ACBD4 PRC1 KCTD20
