# Supplementary material for: Sex-stratified genome-wide association study of multisite chronic pain in UK Biobank
Source: PLoS Genet. 2021 Apr 8;17(4):e1009428. doi: 10.1371/journal.pgen.1009428 (PMC8031124; doi:10.1371/journal.pgen.1009428)
Supplement: S3 Table — (PDF) [file pgen.1009428.s003.pdf]

Gene Symbol	Full Name	Notes	Reference(s): PubMed ID(s)
USP4	Ubiquitin Specific Peptidase 4	encodes a protease that deubiquitinates target proteins e.g. ADORA2A and TRIM21, and is involved in maintaining endoplasmic reticulum. Diseases associated with mutations in this gene include Oculopharyngeal Muscular Dystrophy, and related pathways are TNF signalling and protein metabolism	15494318;21331078
TEX29	Testis Expressed 29	protein coding. No further information	NA
DIAPH3	Diaphanous Related Formin 3	Encodes a member of the formin family, a protein family involved in actin remodeling, regulation of cell movement and adhesion. Mutations in this gene have been associated with autosomal auditory neuropathy 1, and certain types of autosomal dominant deafness - related pathways include GPCR signaling and Rho GTPase signaling.	19457867
GNAQ	G Protein Subunit Alpha Q	Protein coding, gene product binds guanine nucleotides. Mutations in this gene have been associated with dysfunction in platelet activation and aggregation, and with Sturge-Weber Syndrome, and congenital malformations of the capillaries. G proteins are required for the regulation of B-cell selection and prevention of B-cell-dependent autoimmunity. Protein has been found (in vitro) to regulate chemotaxis of bone-marrow derived neutrophils and dendritic cells.	8825633; 23656586; 31324725
FAF1	Fas Associated Factor 1	Protein encoded by this gene involved in the initiation of apoptosis. Diseases associated with mutations in this gene include congenital disorder of deglycosylation and mantle cell lymphomas.	19722279; 26842564; 10462485
AP1G1	Adaptor Related Protein Complex 1 Subunit Gamma 1	Gene encodes a gamma-adaptin protein, a class of adaptin-family protein - adaptins are involved in clathrin-coated vesicle transportation.	9653655; 9733768
C7orf50	Chromosome 7 Open Reading Frame 50	Protein coding gene. Diseases associated with mutations in this gene include Fibrolamellar carcinoma	NA
PHLPP2	PH Domain and Leucine Rich Repeat Protein Phosphatase 2	Encodes a protein involved in regulating Akt and PKC signaling, influencing apoptosis and cell proliferation, including inhibition of cancer cell proliferation. Diseases associated with mutations in this gene include colorectal and bladder cancers, and related pathways include GAB1 signalosome and donwstream signaling events of the B cell receptor.	21986499; 24530606; 17386267; 24675471
GPER1	G Protein -Coupled Estrogen Receptor 1	Encodes a multipass membrane protein that localizes to the membrane of the ER, binds estrogen and is then involved in varying downstream signaling resulting in stimulation of adenylate cyclase and the mobilisation of intracellular calcium. Receptor is involved in a wide range of processes - bone and nervous system development, immune function, metabolism and cognition. Diseases associated with this gene include certain breast cancers and disease of hard tissue in the teeth	9479505; 15705806; 2710976;
RHOT2	Ras Homolog Family Member T2	Encodes a member of the Rho GTPase protein family, localised to outer mitochondrial membrane and involved in trafficking, and may be involved in immune response and inflammation. Diseases associated with	12482879; 23732472; 25761903; 24492963

		mutations in this gene include 3-methylglutaconic aciduria, congenital hypomyelinating neuropathy.	
SPATS2	Spermatogenesis Associated Serine Rich 2	Protein coding gene associated with certain breast cancers.	11944913
SUOX	Sulfite Oxidase	Encodes a protein localised to the intermembrane space in mitochondria. Deficiencies in this protein result in neurological abnormalities, usually fatal	16234925; 17459792; 16475804
BPTF	Bromodomain PHD Finger Transcription Factor	Encodes a protein. Diseases associated with mutations in this gene include certain neurodevelopmental disorders and Legius syndrome.	8975731; 10662542; 16728976;
CRTAC1	Cartilage Acidic Protein 1	Encodes a protein localised to deep zone cartilage, and is a marker distinguishing chrondrocytes from osteoblasts/ mesenchymal stem cells in culture. Protein potentially involved in cell-cell and/or cell-matrix interactions.	11139377;
LRRC39	Leucine Rish Repeat Containing 39	Encodes a protein involved in normal contractile function of the heart. Diseases associated with mutations in this gene include myasthenic syndrome (congenital), and familial hypertrophic cardiomyopathy. function of the heart, and myocyte response to biomechanical stress.	12975309;
NOVA1	NOVA Alternative Splicing Regulator 1	Encodes an RNA-binding protein which is specific to neurons. Diseases associated with this gene include partial fetal alcholo syndrom and low-grade astrocytoma, and the gene protein product may be involved in RNA splicing regulation and metabolism in certain developing neurons.	8558240; 8398153; 9154818; 10719891; 12808107; 16041372; 16713569
TAT	Tyrosine Aminotransferase	Gene encodes a mitochondrial protein, found in cells in liver. Mutations in this gene result in tyrosinemia (Richner-Hanhart syndrome) (disorder involves major skin and corneal lesions, potential cognitive disability). A regulator of this gene is X-linked.	28255985; 27285949; 23954227; 22311600
PPCDC	Phosphopantothenoylcysteine Decarboxylase	Part of an essential universal pathway in eukaryotes and prokaryotes (biosynthesis of CoA from vitamin B5). Associated diseases include Cecum carcinomas.	11923312; 15581364 ; 15450493 ; 16371361
TMEM132B	Transmembrane Protein 132B	Protein coding gene associated with intracranial aneurysm. May also be involved in axonal guidance.	25803036; 27992416 ; 29088312
ITPR3	Inositol 1,4,5-Triphosphate Receptor Type 3	Encodes a receptor for inositol 1,4,5-triphosphate, a second messenger which mediates intracellular calcium release. KO studies in mice indicated that these receptors were involved in exocrine secretion (and so energy metabolism and growth). Associated diseases include diabetes, and certain types of anhidrosis. Also linked to SLE in a Japanese population, in addition to a range of systemic rheumatic diseases.	8081734; 18219441; 17437169
SKIDA1	SKI/DACH Domain Containing 1	Encodes a protein associated with multiple types of cancer, and with domains of unknown function.	23535730; 28585546; 23535730; 21909266
ACBD4	Acyl-CoA Binding Domain Containing 4	Encodes a member of the acyl-CoA binding domain-containing protien family, which bind acyl-CoA thiol esters.	NA

PRC1	Protein Regulator of Cytokinesis 1	Encodes a protein involved in cytokinesis, highly expressed during S and G2/M phases. Protein is a substrate for several cyclin-dependent kinases. Associated diseases include Noonan syndrome and bladder cancers.	9885575; 16756502;
KCTD20	Potassium Channel Tetramerization Domain Containing 20	Encodes a protein that may participate in the AKT-mTOR-p70 S6k signaling cascade, and is a relative of BTBD10, a gene whose protein product is involved in prevention of motor neuronal cell death and accelerating the growth of pancreatic beta cells.	23592240; 24156551
