# Supplementary material for: Sex-stratified genome-wide association study of multisite chronic pain in UK Biobank
Source: PLoS Genet. 2021 Apr 8;17(4):e1009428. doi: 10.1371/journal.pgen.1009428 (PMC8031124; doi:10.1371/journal.pgen.1009428)
Supplement: S4 Table. Expression of genes associated with male MCP in MAGMA analyses across neural and non-neural tissues. TPM = transcripts per million, DRG = dorsal root ganglion. h_DRG_enrich = DRG enrichment score. h_entropy = normalized Shannon’s entropy. h_neural_propn = neural proportion score — (PDF) [file pgen.1009428.s004.pdf]

h_ensembl_id	h_gene_nam	h_DRG_TPI	h_Spinal_C	h_Nucleus_	h_Caudate	h_Hippocar	h_Frontal_(	h_Heart_TF	h_Skeletal_	h_Liver_TP	h_Lung_TP	h_Small_In	h_Whole_B	h_entropy	h_drg_en	h_neural_	Known male expression or function
ENSG00000187323.7	DCC	0.18	6.46	12.18	9.01	9.98	7.49	0.00	0.00	0.00	4.47	5.56	0.03	0.77	0.00	0.82	Role in testicular tumor suppression, >10% DE increase in male brain regions (FC)
ENSG00000128573.17	FOXP2	1.25	3.26	8.98	6.22	3.01	7.30	2.53	4.71	3.51	3.85	72.52	0.19	0.66	0.00	0.24	
ENSG00000164077.9	MON1A	29.55	34.79	23.71	21.86	27.05	30.72	1.23	9.09	1.14	4.16	28.25	8.20	0.91	0.01	0.71	Highest GTEx expression in testis, >10% DE increase in male brain regions like caudate
ENSG00000197183.8	C20orf112	17.14	24.11	17.94	16.91	33.03	19.36	3.90	0.61	4.35	19.53	25.18	21.96	0.93	0.01	0.57	
ENSG00000164068.11	RNF123	21.29	14.18	37.22	27.16	20.99	23.31	9.83	77.33	8.62	28.24	43.10	25.15	0.86	0.01	0.32	
ENSG00000164061.4	BSN	3.73	1.99	19.43	15.12	8.15	25.13	0.11	0.11	0.12	0.26	0.20	0.19	0.67	0.02	0.98	> 10% increase in brain regions like cerebellar hemisphere
ENSG00000145022.4	TCTA	85.26	56.33	56.19	46.98	36.53	43.13	22.49	7.37	18.48	16.69	10.47	22.83	0.94	0.01	0.69	
ENSG00000176095.7	IP6K1	21.01	33.64	41.83	40.90	31.47	44.70	6.26	6.23	3.47	23.10	17.45	32.90	0.94	0.00	0.63	> 10% increase in brain regions like cerebellar hemisphere
ENSG00000183763.4	TRAIP	2.33	3.32	11.25	3.32	5.58	3.47	0.80	0.54	0.50	6.80	6.49	2.32	0.92	0.00	0.59	Highest GTEx expression in testis, >10% DE increase in male brain regions like caudate
ENSG00000001617.7	SEMA3F	10.08	6.33	9.11	8.21	2.95	11.27	0.83	7.17	0.12	38.64	25.27	1.36	0.84	0.02	0.38	
ENSG00000164076.12	CAMKV	0.19	12.07	466.83	280.51	56.07	140.88	0.00	0.00	0.00	0.61	0.00	0.97	0.50	0.00	1.00	> 10% increase in brain regions like nucleus accumbens
ENSG00000004864.8	SLC25A13	6.06	48.13	19.51	11.50	35.81	13.17	11.89	3.13	103.40	30.69	76.43	11.31	0.77	0.00	0.28	
ENSG00000135842.12	FAM129A	5.17	5.83	2.77	2.02	2.20	0.61	219.00	34.23	1.87	28.56	49.70	106.16	0.59	0.00	0.03	
ENSG00000187492.4	CDHR4	0.06	1.33	3.41	7.43	2.43	5.44	0.04	0.00	0.00	7.59	0.00	0.54	0.72	0.00	0.70	
ENSG00000185052.6	SLC24A3	2.08	5.59	35.32	23.57	38.11	16.63	2.01	34.14	0.26	9.23	27.07	2.68	0.80	0.00	0.51	
ENSG00000087269.11	NOP14	11.12	11.36	14.21	14.48	11.58	13.20	14.99	11.27	12.82	28.09	14.05	11.80	0.98	0.00	0.37	
ENSG00000203760.4	CENPW	6.55	1.43	3.03	1.63	6.93	3.54	0.91	0.00	2.01	11.65	2.90	10.87	0.82	0.02	0.38	
ENSG00000125388.14	GRK4	11.88	23.31	23.51	19.90	24.48	34.04	17.89	6.98	2.56	13.49	12.78	6.21	0.96	0.00	0.63	Highest GTEx expression in testis, >10% DE increase in male brain regions like cerebellar hemisphere
ENSG00000161896.6	IP6K3	0.85	8.01	2.88	0.91	10.77	1.59	34.26	33.90	5.53	6.97	0.00	1.42	0.64	0.00	0.16	
ENSG00000003756.12	RBM5	35.71	93.32	126.46	88.43	123.12	89.23	107.97	69.08	93.14	232.82	113.97	113.42	0.97	0.00	0.35	
ENSG00000109919.4	MTCH2	87.11	58.56	71.75	66.96	58.19	57.48	218.15	79.08	492.35	41.11	54.05	41.65	0.73	0.01	0.20	
ENSG00000184056.9	VPS33B	17.92	19.12	27.80	21.55	16.06	26.02	10.39	7.85	14.63	20.01	14.17	20.79	0.98	0.00	0.50	
ENSG00000048828.11	FAM120A	22.11	29.11	36.88	35.67	26.70	30.85	85.99	39.25	57.15	41.34	127.30	18.54	0.92	0.00	0.26	
ENSG00000004534.9	RBM6	35.62	158.96	157.69	218.80	252.72	251.52	124.00	48.61	101.98	553.93	549.77	93.00	0.93	0.00	0.39	
ENSG00000101350.6	KIF3B	11.51	25.11	34.23	37.75	17.97	26.09	8.79	1.48	14.95	8.17	20.61	2.46	0.93	0.00	0.66	
ENSG00000175029.11	CTBP2	16.32	29.08	25.99	33.74	31.95	34.74	22.49	17.37	4.17	37.31	99.56	24.95	0.94	0.00	0.41	
ENSG00000140403.7	DNAJA4	40.67	31.47	78.90	67.43	45.58	60.85	130.11	57.13	2.24	20.16	10.07	11.06	0.87	0.01	0.48	
ENSG00000176020.7	AMIGO3	36.41	3.29	3.33	3.32	1.27	5.40	0.05	3.76	0.18	2.11	0.00	0.28	0.57	0.26	0.84	
ENSG00000173540.8	GMPPB	17.13	8.94	18.52	14.31	13.80	16.26	4.35	9.14	5.03	25.29	23.44	11.82	0.98	0.00	0.47	
ENSG00000171456.12	ASXL1	11.83	26.04	17.34	30.67	47.80	45.34	28.88	37.64	34.22	167.40	310.72	22.27	0.83	0.00	0.21	
ENSG00000114439.13	BBX	5.01	28.40	26.82	18.68	38.15	6.20	17.50	12.17	7.73	24.82	333.28	4.75	0.65	0.00	0.23	
ENSG00000145029.7	NICN1	21.06	28.49	52.48	45.13	47.57	68.01	19.46	15.12	9.83	43.91	9.03	8.57	0.96	0.00	0.64	
ENSG00000173531.10	MST1	8.01	28.30	46.85	58.97	43.61	41.73	0.95	17.09	231.60	59.41	194.54	17.49	0.72	0.00	0.23	
ENSG00000137288.4	MNF1	111.47	93.13	101.71	97.85	65.68	81.34	224.79	91.25	47.94	35.79	42.53	24.35	0.92	0.01	0.44	
ENSG00000164078.7	MST1R	0.55	1.40	1.06	1.46	1.87	3.26	0.57	0.11	0.00	18.08	19.82	1.28	0.65	0.00	0.20	
ENSG00000182179.6	UBA7	43.52	46.49	49.07	37.08	26.76	18.59	12.57	22.60	11.16	233.73	82.95	87.23	0.86	0.01	0.30	
ENSG00000067560.6	RHOA	345.31	361.98	205.22	185.72	209.24	134.78	363.37	183.73	199.31	279.12	176.30	464.95	0.94	0.01	0.37	Overexpression in testicular cancer
