# Supplementary material for: Sex-stratified genome-wide association study of multisite chronic pain in UK Biobank
Source: PLoS Genet. 2021 Apr 8;17(4):e1009428. doi: 10.1371/journal.pgen.1009428 (PMC8031124; doi:10.1371/journal.pgen.1009428)
Supplement: S5 Table — TPM = transcripts per million, DRG = dorsal root ganglion. h_DRG_enrich = DRG enrichment score. h_entropy = normalized Shannon’s entropy. h_neural_propn = neural proportion score. (PDF) [file pgen.1009428.s005.pdf]

h_ensembl_id	h_gene_name	h_DRG_TP	h_Spinal_C	h_Nucleus_	h_Caudate_	h_Hippocar	h_Frontal_C	h_Heart_TP	h_Skeletal_	h_Liver_TPM	h_Lung_TP	h_Small_In	h_Whole_B	h_entropy	h_drg_en	h_neural_	Known female expression or function
ENSG00000196782.7	MAML3	2.20	10.86	5.50	4.39	5.54	5.33	1.30	2.97	1.95	14.07	67.66	9.67	0.76	0.00	0.24	-
ENSG00000143374.9	TARS2	18.41	27.25	35.38	30.01	27.61	29.67	27.50	35.25	14.67	20.72	72.66	11.06	0.96	0.00	0.40	-
ENSG00000143369.10	ECM1	10.45	11.02	6.68	6.99	6.87	12.76	15.07	3.44	6.37	36.98	10.92	2.61	0.93	0.01	0.38	-
ENSG00000146555.14	SDK1	2.86	1.37	1.04	0.94	1.85	1.79	1.13	2.22	0.05	17.82	26.54	0.52	0.68	0.02	0.17	-
ENSG00000187145.10	MRPS21	151.84	83.98	112.73	99.98	85.41	135.00	109.37	24.98	41.49	59.80	50.84	25.21	0.96	0.01	0.61	>10% DE decrease in female brain regions like cerebellar hemisphere
ENSG00000117360.8	PRPF3	23.39	39.97	49.42	40.43	43.57	49.29	49.12	18.45	58.47	91.70	22.31	41.68	0.96	0.00	0.38	-
ENSG00000196821.5	C6orf106	41.04	28.24	53.64	52.95	40.89	57.20	95.97	114.42	39.33	38.02	41.12	22.48	0.90	0.01	0.33	-
ENSG00000124562.5	SNRPC	151.38	102.85	106.97	92.63	63.81	72.08	203.37	71.66	51.60	54.87	39.58	59.10	0.94	0.01	0.46	-
ENSG00000196141.7	SPATS2L	53.94	145.09	88.51	107.52	90.95	156.09	60.72	80.08	67.69	92.64	296.36	5.23	0.94	0.00	0.46	-
ENSG00000065060.12	UHRF1BP1	3.00	1.86	3.33	2.51	1.76	2.08	12.09	5.26	4.95	2.74	3.97	0.87	0.86	0.01	0.25	-
ENSG00000163125.11	RPRD2	3.32	10.21	16.28	14.34	11.20	15.82	15.04	12.70	9.22	9.47	27.67	4.00	0.96	0.00	0.39	-
ENSG00000187323.7	DCC	0.18	6.46	12.18	9.01	9.98	7.49	0.00	0.00	0.00	4.47	5.56	0.03	0.77	0.00	0.82	Associated with agenesis of corpus callosum which is more common in females, >10% DE decrease in female brain regions like frontal cortex
ENSG00000159208.11	C1orf51	9.59	6.52	36.97	29.15	6.33	19.73	22.77	28.57	4.48	4.57	5.18	3.37	0.86	0.01	0.47	-
ENSG00000144290.9	SLC4A10	0.16	4.78	23.23	9.81	30.64	42.15	0.00	0.00	0.50	0.14	0.29	0.24	0.61	0.00	0.98	>10% DE increase in female brain regions like anterior cingulate cortex
ENSG00000181163.9	NPM1	371.85	455.55	394.71	314.22	316.30	235.59	700.44	419.61	331.87	495.37	405.32	190.16	0.96	0.00	0.37	-
ENSG00000010810.11	FYN	89.79	210.35	355.69	317.59	271.16	291.79	48.16	13.96	32.98	216.83	150.98	113.48	0.94	0.00	0.68	Highest GTEx tissue expression in ovary, related to oocyte development
ENSG00000145864.8	GABRB2	0.02	2.08	22.23	13.26	16.51	45.03	0.06	0.00	0.00	0.53	9.82	0.13	0.64	0.00	0.90	>10% DE decrease in female brain regions like frontal cortex
ENSG00000021826.10	CPS1	0.46	13.03	2.25	5.64	4.19	3.63	2.47	1.34	2137.00	2.80	992.46	0.06	0.21	0.00	0.01	-
ENSG00000129351.13	ILF3	49.58	119.89	118.31	121.56	145.40	160.17	51.54	118.92	22.59	209.14	204.73	48.82	0.96	0.00	0.46	-
ENSG00000116580.13	GON4L	6.72	13.53	24.43	23.10	27.20	28.91	18.44	10.50	22.99	62.20	158.05	16.21	0.87	0.00	0.27	-
ENSG00000176887.5	SOX11	0.83	1.58	3.05	2.21	1.85	0.94	0.42	0.04	0.00	0.01	0.00	0.00	0.75	0.02	0.94	Related to poor prognosis in breast cancer
ENSG00000129353.10	SLC44A2	170.02	216.56	300.82	259.89	209.15	162.66	69.60	387.72	6.46	409.00	141.68	671.10	0.84	0.01	0.33	-
ENSG00000183049.8	CAMK1D	16.82	14.96	68.27	61.48	27.47	66.92	1.08	1.57	2.53	24.58	70.99	68.62	0.84	0.01	0.52	Role in breast cancer, >10% DE increase in female brain regions like amygdala
ENSG00000148219.12	ASTN2	44.35	24.30	46.33	51.45	65.39	63.86	2.34	4.85	2.12	9.11	26.79	4.56	0.88	0.02	0.83	>10% DE decrease in female brain regions like cerebellar hemisphere
ENSG00000164535.9	DAGLB	15.23	12.72	34.44	31.25	21.14	29.39	2.83	15.24	2.65	42.96	50.67	25.12	0.94	0.00	0.45	-
ENSG00000078403.12	MLLT10	10.54	11.20	18.75	21.64	73.40	60.00	28.39	11.83	19.48	59.13	253.41	50.06	0.84	0.00	0.28	-
ENSG00000105726.11	ATP13A1	25.77	39.17	62.19	43.66	54.52	53.40	8.29	20.00	6.08	92.25	22.37	45.23	0.94	0.00	0.52	2nd highest GTEx tissue expression in ovary
ENSG00000109436.7	TBC1D9	44.35	6.08	11.31	8.46	5.54	13.04	10.60	0.45	7.07	10.22	14.36	1.71	0.85	0.05	0.63	>10% DE decrease in female brain regions like cerebellar hemisphere, Related to poor prognosis in breast cancer
ENSG00000143702.11	CEP170	15.71	33.45	20.06	15.31	23.21	11.45	23.10	10.39	7.87	20.38	8.78	4.59	0.96	0.00	0.54	>10% DE decrease in female brain regions like cerebellar hemisphere
ENSG00000130287.7	NCAN	1.62	3.32	69.38	85.75	12.76	62.84	0.00	0.00	0.01	0.06	0.00	0.12	0.55	0.00	1.00	>10% DE decrease in female brain regions like c putamen, ~10% increase in female amygdala
ENSG00000118007.8	STAG1	5.72	9.22	7.54	10.64	11.43	10.68	17.95	7.41	12.76	16.00	67.81	9.63	0.90	0.00	0.26	-
