# Supplementary material for: Sex-stratified genome-wide association study of multisite chronic pain in UK Biobank
Source: PLoS Genet. 2021 Apr 8;17(4):e1009428. doi: 10.1371/journal.pgen.1009428 (PMC8031124; doi:10.1371/journal.pgen.1009428)
Supplement: S6 Table — (PDF) [file pgen.1009428.s006.pdf]

ensg	entrezID	symbol	hgnc_symbol	OMIM	uniprotID	DrugBank
ENSG00000255307	255725	OR52B2	OR52B2	NA	Q96RD2	NA
ENSG00000187323	1630	DCC	DCC	120470	P43146	NA
ENSG00000185052	57419	SLC24A3	SLC24A3	609839	Q9HC58	NA
ENSG00000161904	221496	LEMD2	LEMD2	616312	Q8NC56	NA
ENSG00000187609	54932	EXD3	EXD3	NA	Q8N9H8	NA

FUMA GENE2FUNC results (gene table) for male MCP GWAS
