# Supplementary material for: Sex-stratified genome-wide association study of multisite chronic pain in UK Biobank
Source: PLoS Genet. 2021 Apr 8;17(4):e1009428. doi: 10.1371/journal.pgen.1009428 (PMC8031124; doi:10.1371/journal.pgen.1009428)
Supplement: S7 Table — (PDF) [file pgen.1009428.s007.pdf]

ensg	entrezID	symbol	hgnc_symbol	OMIM	uniprotID	DrugBank
ENSG00000185104	11124	FAF1	FAF1	604460	Q9UNN5	NA
ENSG00000123080	1031	CDKN2C	CDKN2C	603369	P42773	NA
ENSG00000118298	23632	CA14	CA14	604832	Q9ULX7	DB00819:DB00909:DB08846
ENSG00000117362	51107	APH1A	APH1A	607629	Q96BI3	DB05171
ENSG00000118292	79630	C1orf54	C1orf54	NA	Q8WWF1	NA
ENSG00000159208	148523	C1orf51	C1orf51	615782	Q8N365	NA
ENSG00000187145	54460	MRPS21	MRPS21	611984	NA	NA
ENSG00000117360	9129	PRPF3	PRPF3	607301	O43395	NA
ENSG00000163125	23248	RPRD2	RPRD2	614695	Q5VT52	NA
ENSG00000143374	80222	TARS2	TARS2	612805	Q9BW92	DB00156
ENSG00000143369	1893	ECM1	ECM1	602201	Q16610	NA
ENSG00000143382	54507	ADAMTSL4	ADAMTSL4	610113	Q6UY14	NA
ENSG00000225996	NA	AL356356.1	NA	NA	NA	NA
ENSG00000154975	56934	CA10	CA10	604642	Q9NS85	DB00909
ENSG00000176887	6664	SOX11	SOX11	600898	P35716	NA
ENSG00000153064	55024	BANK1	BANK1	610292	Q8NDB2	NA
ENSG00000138821	64116	SLC39A8	SLC39A8	608732	Q9C0K1	NA
ENSG00000181163	4869	NPM1	NPM1	164040	P06748	DB11638
ENSG00000156427	8817	FGF18	FGF18	603726	O76093	NA
ENSG00000196821	64771	C6orf106	C6orf106	612217	Q9H6K1	NA
ENSG00000124562	6631	SNRPC	SNRPC	603522	P09234	NA
ENSG00000065060	54887	UHRF1BP1	UHRF1BP1	NA	Q6BDS2	NA

FUMA GENE2FUNC results (gene table) for female MCP GWAS
