# Supplementary material for: Sex-stratified genome-wide association study of multisite chronic pain in UK Biobank
Source: PLoS Genet. 2021 Apr 8;17(4):e1009428. doi: 10.1371/journal.pgen.1009428 (PMC8031124; doi:10.1371/journal.pgen.1009428)
Supplement: S8 Table — *Full results (chip, PCs) not shown for brevity. SE = standard error, Z = Z value, P = p value, OR = odds ratio, PRS = z-standardised PRS value. (PDF) [file pgen.1009428.s008.pdf]

| | Beta | SE (Beta) | Z | P | OR |
|---|---|---|---|---|---|
| (Intercept) | -20.21 | 2.29 | -8.85 | 9.14E-19 | 1.67E-09 |
| Age | 0.0157 | 0.0033 | 4.69 | 2.74E-06 | 1.016 |
| PRS | 0.0026 | 0.0003 | 8.57 | 1.06E-17 | 1.0026 |

Association between male-specific MCP PRS and CWP in men. *Full results (chip, PCs) not shown for brevity. SE = standard error, Z = Z value, P = p value, OR = odds ratio, PRS = z-standardised PRS value.
