# Supplementary material for: Sex-stratified genome-wide association study of multisite chronic pain in UK Biobank
Source: PLoS Genet. 2021 Apr 8;17(4):e1009428. doi: 10.1371/journal.pgen.1009428 (PMC8031124; doi:10.1371/journal.pgen.1009428)
Supplement: S9 Table — *Full results (chip, PCs) not shown for brevity. SE = standard error, Z = Z value, P = p value, OR = odds ratio, PRS = z-standardised PRS value. (PDF) [file pgen.1009428.s009.pdf]

	**Beta**	**SE (Beta)**	**Z**	**P**	**OR**
(Intercept)	-30.65	2.13	-14.41	4.80E-47	4.90E-14
Age	0.0165	0.0029	5.74	9.73E-09	1.0167
PRS	0.0034	0.0002	14.53	7.82E-48	1.0034

Association between female-specific MCP PRS and CWP in women. *Full results (chip, PCs) not shown for brevity. SE = standard error, Z = Z value, P = p value, OR = odds ratio, PRS = z-standardised PRS value.
