# Supplementary figures and images for: Sex-stratified genome-wide association study of multisite chronic pain in UK Biobank
Source: PLoS Genet. 2021 Apr 8;17(4):e1009428. doi: 10.1371/journal.pgen.1009428 (PMC8031124; doi:10.1371/journal.pgen.1009428)

-log<sub>10</sub> P-value

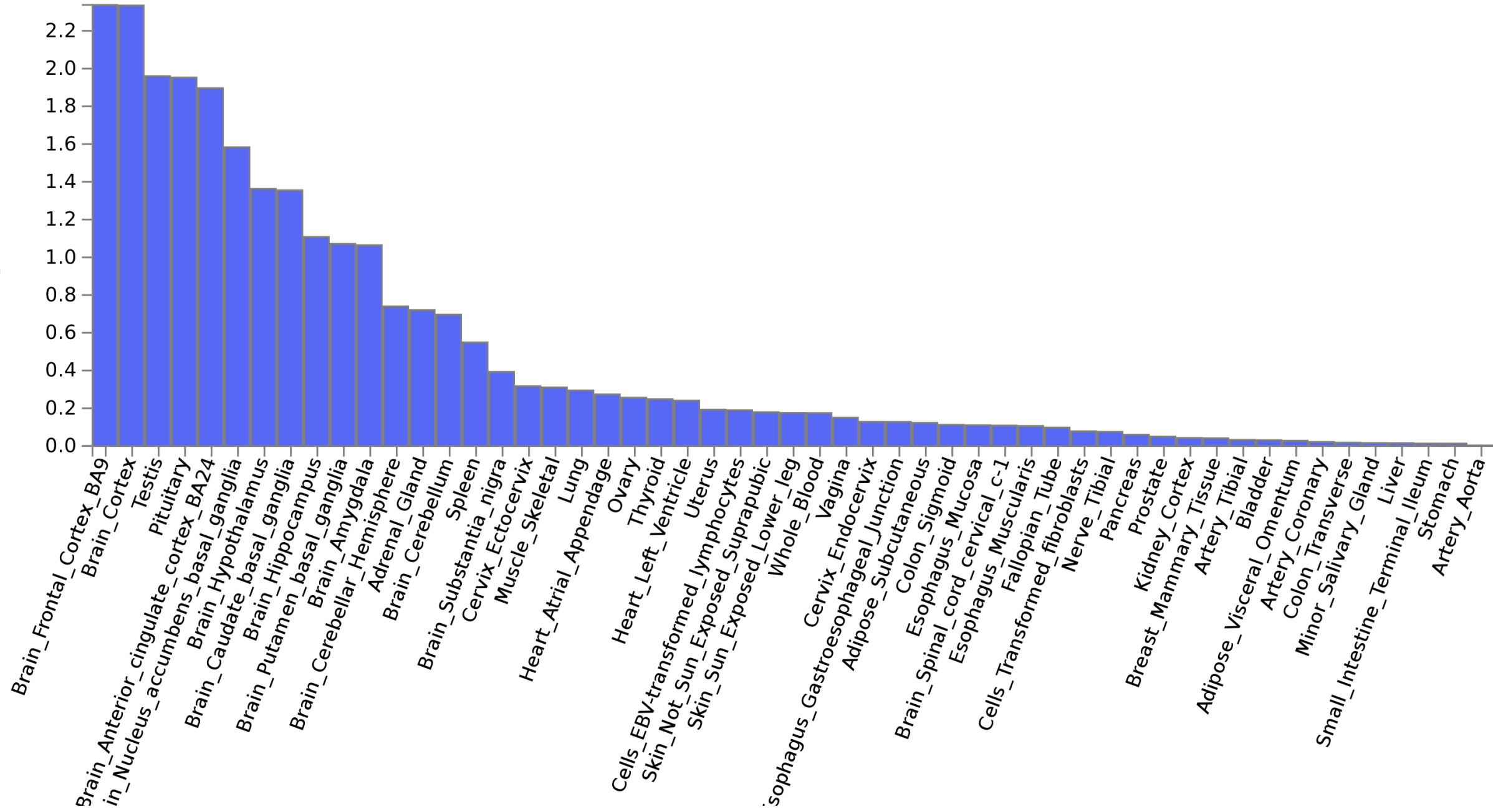

Supplement: S1 Fig — (PDF) [file pgen.1009428.s010.pdf]

-log<sub>10</sub> P-value

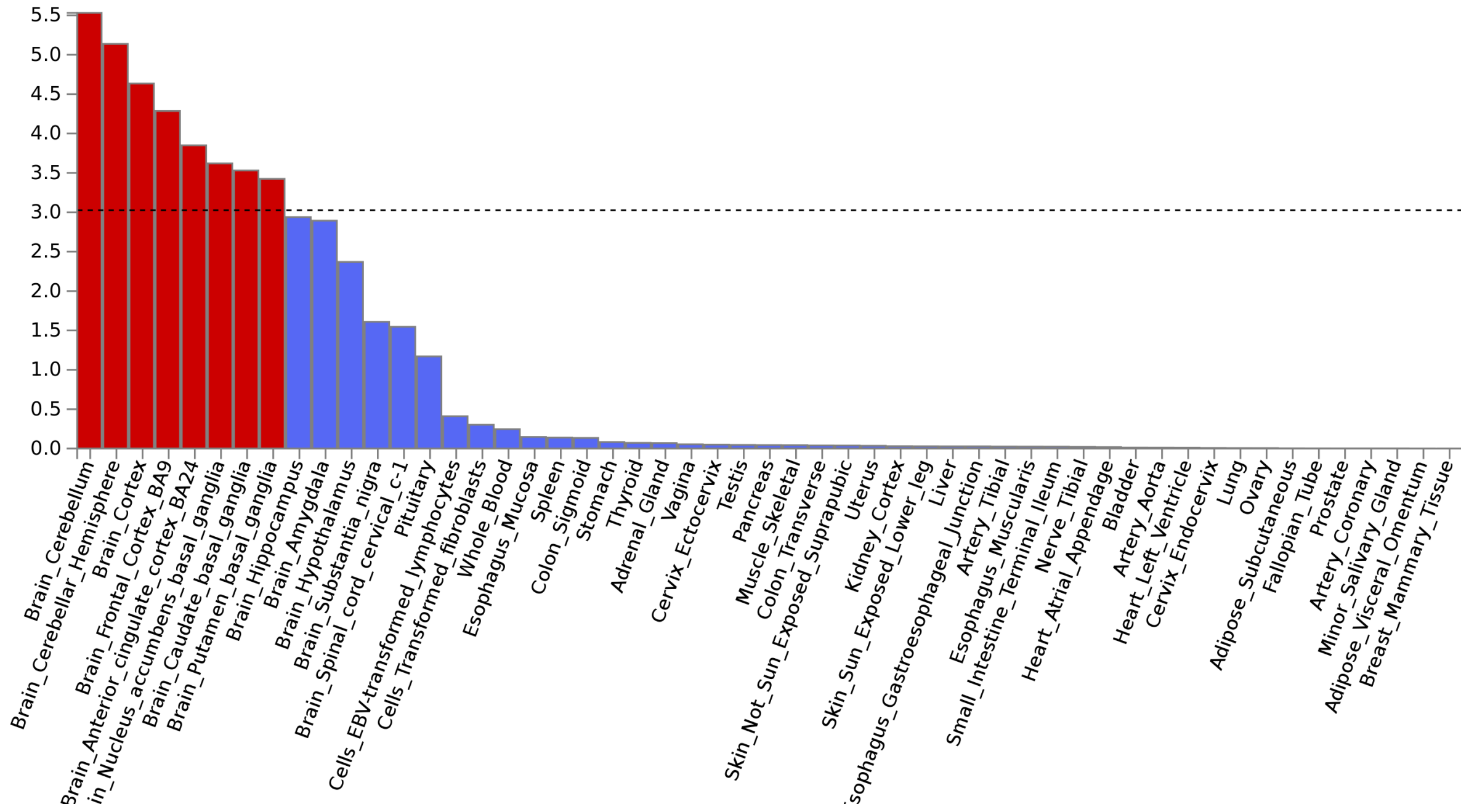

Supplement: S2 Fig — (PDF) [file pgen.1009428.s011.pdf]

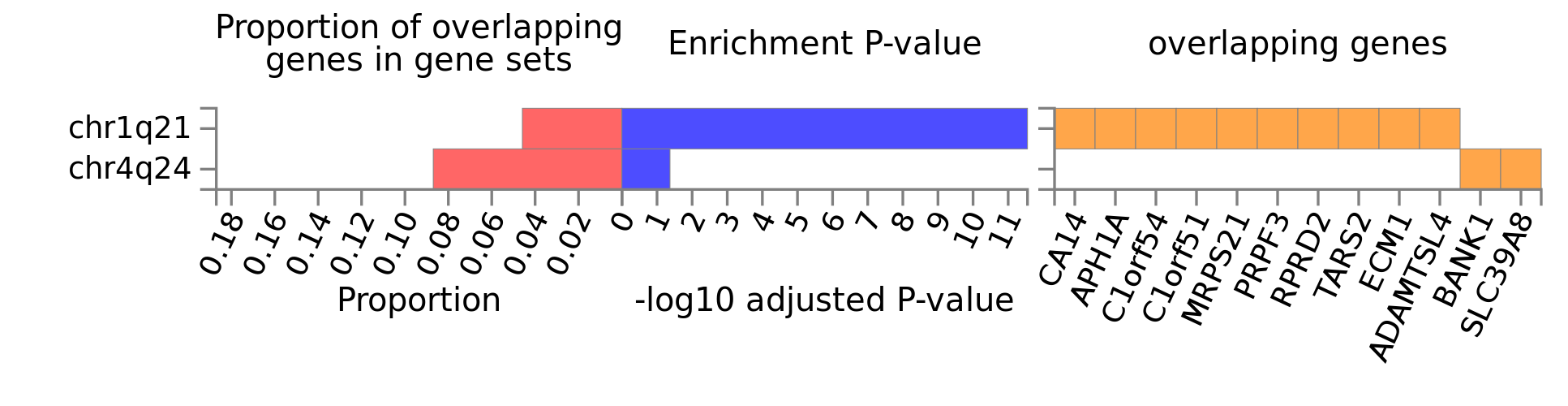

Supplement: S3 Fig — (TIFF) [file pgen.1009428.s012.tiff]
